# Optimized Position Weight Matrices in Prediction of Novel Putative Binding Sites for Transcription Factors in the *Drosophila melanogaster* Genome

**DOI:** 10.1371/journal.pone.0068712

**Published:** 2013-08-06

**Authors:** Vyacheslav Y. Morozov, Ilya P. Ioshikhes

**Affiliations:** 1 Department of Mathematics and Statistics, University of Ottawa, Ottawa, Canada; 2 Ottawa Institute of Systems Biology, Ottawa, Canada; 3 Department of Biochemistry, Microbiology and Immunology, University of Ottawa, Ottawa, Canada; University of Maryland School of Medicine, United States of America

## Abstract

Position weight matrices (PWMs) have become a tool of choice for the identification of transcription factor binding sites in DNA sequences. DNA-binding proteins often show degeneracy in their binding requirement and thus the overall binding specificity of many proteins is unknown and remains an active area of research. Although existing PWMs are more reliable predictors than consensus string matching, they generally result in a high number of false positive hits. Our previous study introduced a promising approach to PWM refinement in which known motifs are used to computationally mine putative binding sites directly from aligned promoter regions using composition of similar sites. In the present study, we extended this technique originally tested on single examples of transcription factors (TFs) and showed its capability to optimize PWM performance to predict new binding sites in the fruit fly genome. We propose refined PWMs in mono- and dinucleotide versions similarly computed for a large variety of transcription factors of *Drosophila melanogaster*. Along with the addition of many auxiliary sites the optimization includes variation of the PWM motif length, the binding sites location on the promoters and the PWM score threshold. To assess the predictive performance of the refined PWMs we compared them to conventional TRANSFAC and JASPAR sources. The results have been verified using performed tests and literature review. Overall, the refined PWMs containing putative sites derived from real promoter content processed using optimized parameters had better general accuracy than conventional PWMs.

## Introduction

Transcription Factors (TFs) play a crucial role in gene regulation, usually binding to DNA through recognizing certain motifs in one or two strands of DNA adjacent to the regulated gene. The prediction of TF binding sites (TFBSs) is a big challenge for computational biologists, particularly as increasing amounts of sequence data become available. Direct experimental investigations of TF-DNA binding are still rather time-consuming and labour-intensive.

Position weight matrices (PWMs) became essential computational tool and model of choice to describe sequence binding specificities of particular TF-DNA interactions. The relatively short (5–15 nt) sequence motifs are recognized by TFs whose sequence specificities are not very strict [1], [2]. In addition, the laboratory *in vitro* measurements show a variability of affinity for TF-DNA binding which results in a potentially large number of binding sites in genome [3]. The variability in the binding sites of a single factor and molecular mechanisms underlying these variations are not well understood.

PWMs are usually generated from a set of functionally related and aligned TF binding sites, and there are several reported approaches to this task. We use PWMs constructed from log-odds ratios of occurrences of mono- or dinucleotides with taking into account background nucleotide distributions. Gershenzon et al. argued that known PWMs suffer from low specificity and sensitivity when used as a prediction tool to discover putative binding sites [4]. Computational biologists developed a plethora of improvements to provide more complete models of TFBSs making a variety of assumptions, but problems of binding specificity and TFBS prediction remain far more complex to be completely resolved. Recently Stormo and Zhao confirmed that many of the existing approaches are not accurate or complete in depicting binding specificity or how well a protein can distinguish between different sequences [3].

In addition to limited knowledge of binding specificity incorporated into the matrices, the mononucleotide PWMs assume that the nucleotides of the binding sites exert independent effects on the binding affinity. Dinucleotide matrices provide more reliable models considering the presence of Markovian properties in the binding nucleotide sequences [5]. However, providing a better description, the dinucleotide model was applied to only few particular TFs and did not address the binding motif specificity for a variety of discovered TFs. While our earlier publication [4] provided a proof of principle for the suggested algorithm with application to a single transcription factor (Sp1), it was not implemented as a genomic scale application for a variety of TFs. The present work represents the first large scale study of cooperative influence of adjacent nucleotides onto TF-DNA binding by calculating optimized PWMs (mono- and dinucleotide alike) for a variety of transcription factors from *Drosophila*. The analysis was made not for an arbitrary selected TF, but for all available TF, including so called [11] law quality matrices.

The *in situ* approach to accurately defining the DNA-binding sequence specificity of transcription factors would rely upon a representative and complete collection of binding sites from properly designed laboratory experiments, such as SELEX [6], protein binding microarrays [7], or *in-vivo* ChIP-Seq data. *In silico* detection of weak protein-binding signals with up to one nucleotide resolution in noisy ChIP-Seq data remains to be challenging [8], [9]. In practical applications an alternate confirmation of TFBS functional relevancy is a necessary step in any ChIP experiment [10]. The *in silico* approaches to determining binding specificity are far less expensive, although their common limiting factor is a number of validated binding sites. The knowledge of binding specificity for most TFs is not complete, particularly, the knowledge of variability of binding sequences.

To improve existing matrix-based models we chose to use the commercial TRANSFAC database [11] complemented by JASPAR [12] for two reasons: 1) TRANSFAC assures an expert-curated experimentally confirmed comprehensive set of TFBSs for each TF entry and 2) TRANSFAC supplies each TF entry with a frequency matrix which can be used for prediction of new sites and computing refined matrices. In addition, TRANSFAC is supplied with Match™ tool, which we used as a conventional instrument for TFBS prediction for comparison with our approach.

To cover a bigger number of TFs for *Drosophila melanogaster* (further *Drosophila*) we primarily assigned JASPAR the role of the testing set in contrast to much larger TRANSFAC set used for matrix training.

The main goal of this study is to optimize predictive performance of the matrices published for *Drosophila* in the TRANSFAC database where binding sequences were also available. This selection enabled us to optimize both mono- and dinucleotide matrices by implementing the previously introduced approach of iterative PWM refinement [4], [13].

A key feature of our study consists of drawing sequences with similar composition of nucleotides from an area of promoters with most significant over-representation of the signals. The iterative PWM refinement [4] followed by supervised sampling of new nucleotide sequences can be presented as a heuristic PWM optimization using machine learning [14]. The target function of such optimization is the Matthews correlation coefficient (see Equation 6), which enables the trade-off between the false positive and false negative rates among the observed and predicted sequences [15]. TRANSFAC's collection of TFs with known binding sequences in *Drosophila* has been evaluated to calculate refined PWMs which show biological relevancy and better predictive performance compared to conventional matrices.

## Results

### A quantitative outline of refined PWMs

We optimized 37 TRANSFAC matrices (33 TRANSFAC TF entries), both for mono- and dinucleotide versions shown in Table 1. Refinement of each new matrix in terms of Matthews correlation coefficient *Cor* was done for the parameters such as motif length, location on the promoters and PWM matching cut-off.

**Table 1:** Summary of the statistics for the putative binding sites discovered for TFs published in TRANSFAC™.

	Matrices published in TRANSFAC™				Predicted sites	
##	Matrix AC	TF name	S. Avail.	S.Used∶L	mono found∶L	di found∶L
1	M01083	Abd-A	40	40∶10	209∶8	801∶8
2	M01094	Abd-B	7	7∶7	39∶7	155∶6
3	M00171	Adf-1	7	7∶21	15∶21	69∶20
4	M01095	AP	14	14∶8	768∶7	232∶8
5	M01096	Brk	10	10∶7	57∶8	51∶8
6	M01097	CAD	13	13∶10	408∶9	41∶9
7	M01087	C/EBP	12	12∶23	27∶22	26∶22
8	M00120	Dl	13	13∶11	3∶10	28∶12
9	M00488	DREF	10	10∶13	60∶12	13∶12
10	M00110	Elf1	5	5∶16	166∶13	8∶16
11	M00696	En	11	11∶7	1092∶6	161∶7
12	M00396	En-1	10	10∶7	84∶7	142∶8
13	M00020	Ftz	9	9∶12	26∶14	95∶11
14	M00022	Hb	16	16∶10	1082∶9	341∶11
15	M00021	Kr_01	6	6∶10	427∶9	21∶9
16	M01089	Kr_Q6	30	30∶12	34∶10	29∶10
17	M01090	Mad	9	9∶8	83∶7	152∶8
18	M00487	mtTFA	11	9∶10	262∶9	25∶10
19	M00461	Ovo_01	21	21∶15	17∶12	28∶13
20	M01101	Ovo_Q6	9	9∶8	791∶7	36∶8
21	M01091	PRD	9	9∶7	115∶7	92∶8
22	M01102	SD	19	19∶7	518∶7	277∶6
23	M01098	CF1A	11	11∶16	135∶15	94∶11
24	M00112	CF1	38	38∶9	26∶9	78∶10
25	M00044	Sn	9	9∶14	28∶14	42∶11
26	M00060	Sn	40	12∶13	253∶10	18∶12
27	M00060	Sn	40	22∶10	58∶9	36∶11
28	M00666	Sry-beta	5	5∶9	44∶11	57∶11
29	M00234	Su(H)	10	10∶13	68∶12	118∶12
30	M01092	TCF_Q6	25	25∶16	89∶13	16∶15
31	M00679	Tll	10	10∶8	44∶7	355∶6
32	M01103	TWI	11	11∶14	24∶12	8∶14
33	M00018	Ubx	88	48∶10	1476∶5	535∶7
34	M00283	Z	24	24∶11	92∶9	31∶11
35	M01099	KNI	32	32∶18	91∶17	35∶15
36	M00016	E74A	14	14∶12	120∶13	109∶14
37	M01088	DEAF1	22	22∶7	284∶7	130∶7

Matrix AC is AC (Accession Code) field from TRANSFAC™ (naming preserved); “S. Avail.” and “S. Used” mean respectively a number of sites available in the database and used for matrix training; L and numbers in front and after columns show the number of detected sites and their length (L) respectively for mononucleotide (“mono”) and dinucleotide (“di”) optimized matrices with optimized cut-off.

We found that improved matrices generated by the proposed refinement technique perform better than conventional TRANSFAC matrices (initial PWMs). For this analysis, we used a summary of predictions on synthetic tests (see Table S1 in File 1) and a measure of information content presented as sequence Logos for discovered putative TFBSs (Figure S1 and other figures in Files S2 and S3). The refined PWM in mono and dinucleotide formats can be found in File S4.

A more comprehensive analysis, including comparison with Match™ tool and validation of a biological content of some of our findings, is performed in the following sections.

The length of the initial matrices is equal to the common part of aligned TFBSs. TRANSFAC sites in most cases have already been aligned in the database. However, the resulting mononucleotide PWMs did not necessarily preserve the initial lengths. This happens because the matrix refinement procedure offers the motif length to find matches with largest *Cor*. For this reason, we can observe shifts in positioning toward the most conserved nucleotides within the initial sequence, which accounts for the irregularity of background nucleotide distribution on promoter sequences used for training. In particular, for Brk, Ftz, Dl, En, Hb, one or both optimized matrices have longer lengths than the initial sequences.

As seen from Table 1 or Table S1, for each particular TF, the averaged total number of discovered sites is neither for real nor for synthetic data larger than the total number of promoter sequences used for PWM training. Employing an arbitrary lower cut-off value with the original PWM would apparently produce more matches, most of which would be false positives. Low specificity, which hampers the application of conventional PWMs for TFBS prediction [16], is therefore overcome by our approach. Taking into account our limited knowledge, some optimized matrices can be expected with better or worse (when compared to the original PWMs) specificity or sensitivity in a single analysis. Someone interested in a simultaneous application of many PWMs can benefit from a generally better total performance of the optimized matrices. In particular, result of the comparison presented in Table S1 provides extra insight into the differences between optimized mono- and dinucleotide matrices when they are applied to a completely new promoter content (synthesized test sequences) that was not used in the matrix training. In the next section we will give more comprehensive quantitative analysis of PWM tests, but here we provide a big picture of the predictive performance of our method.

The following selected results come from analysis of one of synthetic tests constructed from biological binding sequences taken from JASPAR. For comparison, since TFs have different total numbers of true positive (TP) and false positive (FP) matches, we characterized the estimated sensitivity and specificity through proportions of all predicted TP (or FP) to the size of a respective test set. To observe results from a big picture, all TP and FP matches discovered in JASPAR synthetic tests (Table S1) were collected by the type of matrix in three groups (Initial, OPT mono, OPT di). In total, the initial matrices give 77 true positives against 127 false positives; optimized mononucleotide matrices give 123 true positives against 73 false positives; finally, optimized dinucleotide matrices give 106 true positives against 76 false positives. Because both optimized mono- and dinucleotide matrices were tested on the same testing sets, we can easily calculate the percentage of improvement when using optimized matrices against the initial matrices calculated from TRANSFAC.

The total number of TPs improved (increased) by 59.7% (out of total 77) for the mononucleotide matrices and by 37.7% for the dinucleotide matrices compared to the result from initial matrices used in TRANSFAC. Similarly, for the number of FPs a global picture comes as follows: for the mononucleotide PWMs the total number of FPs decreased by 42.5% (out of total 127) and for the dinucleotide matrices it decreased by 40.2%. The comparison was done with a fixed cut-off of the corresponding optimized matrix. The analysis of the results in terms of common statistical precision – recall indicators also showed better predictive performance of the optimized matrices over the Match™ tool predictions and is presented in section “Optimized PWMs show an increased accuracy in simulations”.

**Figure 1:**
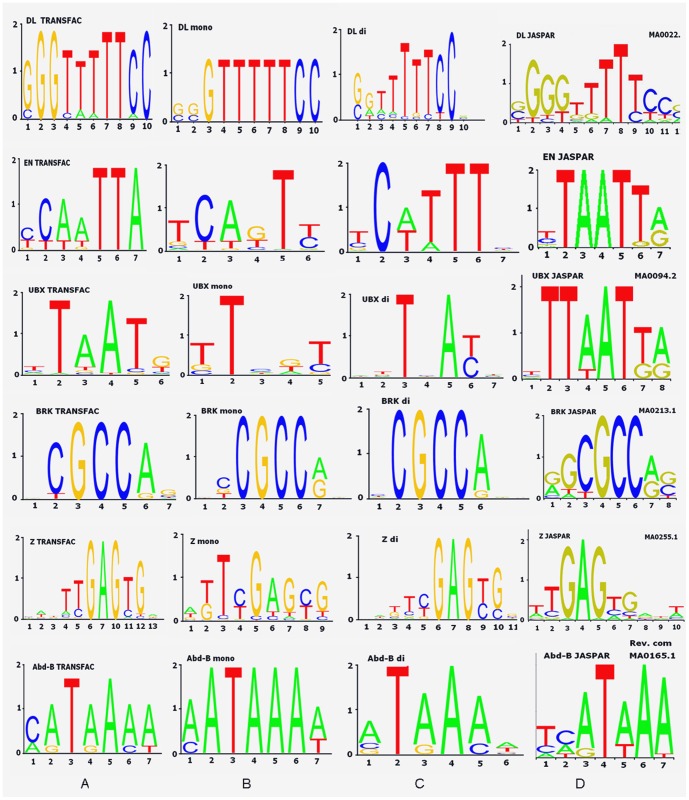
Information content of new sites found by the optimized matrices compared to TRANSFAC™ and JASPAR sites. (A) Initial TRANSFAC™ sites. (B) From new discovered sites (mononucleotide matrix). (C) From new discovered sites (dinucleotide matrix). (D) From JASPAR CORE collection. Shown sequence Logo for Dl, En,Ubx,Brk, Z and Abd-B TFs.

Sequence Logos in Figure 1 show how well nucleotide content is conserved in the putative binding sequences found here. The sequence Logo is constructed from information content [17] measured in bits and shows the consensus sequence along with relative frequency of bases at every position of a binding site. For reference, the Logo of the initial TRANSFAC sequences was included in the first column. Putative TFBSs newly discovered by the mono- and dinucleotide optimized matrices with the optimal cut-offs are placed in the second and third columns respectively. PWM refinement method is not an exhaustive method for identifying the overrepresented motifs in promoter sequences. Each selected TF entry (rows in Figure 1) emphasizes similarity of consensus patterns between Logos from columns A, B, C, D. Sequence Logos for the remaining TFs are placed in the pages 1–3 [see File S2] and pages 1–2 [see File S3], and are presented by only columns A,B,C (there was no JASPAR entries to compare).

The prevalence of the most conserved nucleotides as the tallest letters in the Logos (Figure S1 and other Figures from Files S2 and S3) for some TFs does not necessarily follow the arrangement presented in the initial consensus. The first two or rarely three top nucleotides sometimes are permuted or changed. For instance, that happens at positions 5 and 11 in Z, positions 1, 4, 7 in En, and position 1 in Abd-B (positions numbered in left-to-right order as shown in Figure 1 with reference to position numbers in column A). This variability might indicate nucleotide positions with significant degeneracy. Other examples can be found in sequence Logos from Files S2 and S3, which also include Logos of discovered sites for other TFs. From such examples we can see that often the initial consensus pattern was reproduced at the top Logo nucleotides although with a lower entropy. The positions where this happened should be considered more stable than positions where swapping has occurred. More details on this matter are provided in the Discussion.

In addition to that, our results (Figure 1) show that some nucleotide positions in putative sites appeared to be more conserved by the information content [17] than expected from initial sequences, for instance: Dl at positions 4, 5, 6; En at positions 1, 2, 7; Brk at position 2, and Abd-B at positions 2, 4, 6 (all positions related to the initial sequence consensus starting from left to right).

Inspection of the sequence Logos from predicted TFBS sequences on the aligned promoters shows that motifs discovered using dinucleotide matrices are in general more similar to those used in training and thus are likely biased by the content of initial sequences. This can be seen when comparing top nucleotides from most respective Logos in columns C to A vs. B to A in Figure 1 with the exception of Dl, which might demand more special investigation. We suggest (without estimating the quantitative effect, which is beyond the scope of the current study) that this visible property might result from taking into account the co-occurrence of neighboring nucleotides during dinucleotide matrix optimization.

Besides the better predictability on synthetic tests, the refined PWMs with optimized motif length and cut-off show biological relevancy on real promoter sequences.

### Biological relevancy of the motifs discovered by optimized PWMs

*In silico* identification of TFBSs requires independent confirmation of the results by alternative approaches. To investigate whether motifs found by our analysis are biologically relevant, we used the UniPROBE database, which is generated by protein binding microarrays (PBM) for a range of proteins (406 at the time of analysis) from different organisms (currently not including *Drosophila*) [7], [18]. Consequently the UniPROBE database is relatively accurate collection of data on TF-DNA binding [18].

Each protein entry in UniPROBE provides quantitative preferences for all possible nucleotide sequence variants (“words”) of length k (called “k-mers”). The small number of proteins collected in UniPROBE prevents us from performing a comprehensive annotation of all putative TFBSs identified using refined PWMs. Thus, we used UniPROBE to illustrate capabilities of the optimized PWMs to predict new biologically meaningful TFBSs. As such we were able to map some of our putative binding sites to known orthologous TFs that had similar DNA binding preferences based on the data in UniPROBE.

For this analysis we selected non-redundant putative TFBSs of three arbitrary TFs: Ubx, En and TCF which were cleared from redundant sites (further we refer to the selected sites as PWM-sites for brevity). Ubx and En are homeodomain proteins and homeodomains have conserved amino acid sequences across most of eukaryotic organisms. This high degree of conservation makes them an ideal system for studies attempting to elucidate specific protein-DNA interactions. The third TF was TCF (Pangolin) with High Mobility Group box (HMG-box) sequence specific binding domain. Given the evolutionary conservation of both binding sites and protein sequences, we reasoned that these three sets of PWM-sites would be similar to known motifs for other binding proteins with similar DNA binding domain structures, and that finding such a similarity would provide additional support that matched new PWM-sites are likely to represent genuine TFBSs.

We used the UniPROBE search tool (available at http://the_brain.bwh.harvard.edu/uniprobe/) with default settings and queried putative PWM-sites for Ubx, En and TCF on the promoter data set to filter out TFs that bind similar DNA (oligonucleotide) motifs in other organisms. The upper boundary for E-values was set to 0.001, which corresponded to E-values returned from queried original sites from TRANSFAC. The E-value is utilized as a ranking tool to select a protein with a known binding site most similar to the submitted TFBS. In total, we queried UniPROBE with 296, 39 and 16 putative non-redundant TFBSs for Ubx, En and TCF, respectively. The numbers correspond to the total number of detected non-redundant PWM-sites for these TFs.

In the remaining part of this section we analyze the similarity between top UniPROBE matches to those three TFs. We used a conventional sequence alignment tool and graphical representation of aligned amino acid sequences to indicate the location of protein binding domains, using existing data sources. Our findings are supported by publications and databases regarding the binding property of these proteins and their closest homologues.

Querying UniPROBE with PWM-sites for Ubx yielded 24 proteins, the PWM-sites for En yielded two, and the PWM-sites for TCF yielded only one. Table 2 lists the top matches from this analysis according to their E-values. Notably, each of the matched TFs CEH-22 and Sox-4 appeared in UniPROBE query report several times with E-values lower than those for original TFBS. From this result we can assume that En and TCF have closer binding specificities with CEH-22 and Sox-4 TFs than with others in UniPROBE.

**Table 2:** Three examples of UniPROBE queries with putative PWM-sites, which were discovered using optimized PWMs.

	Ubx			En			TCF Q6	
Similar motif	Organism	Best E-val.	Similar motif	Organism	Best E-val.	Similar motif	Organism	Best E-val.
Hoxa-6	Mm	0.003571	CEH-22	Ce	0.000305	Sox-4	Hs	0.008115

Only the best matches shown for three tested TF Ubx, En and TCF. Abbreviations: “Ce” means *C. elegans*; “Sc” means *Saccharomyces cerevisiae*; “Mm” means *Mus musculus*; “Hs” means *Homo sapiens*.

We hypothesized that those proteins identified by the UniPROBE analysis were related in terms of the amino acid sequences of their DNA-binding domains. To test this possibility we aligned the amino acid sequences of retrieved proteins using ClustalW2 (available at http://www.ebi.ac.uk/Tools/msa/clustalw2). The aligned amino acid sequences were retrieved from the UniProt database (although the name is similar, this is not the UniPROBE database), available at www.uniprot.org. When UniProt protein had several isoforms, the protein sequence labeled as “canonical” was retrieved. The results were visually assessed with JalView available online with ClustalW2. A standalone JalView application is available at http://www.jalview.org/.

**Figure 2:**
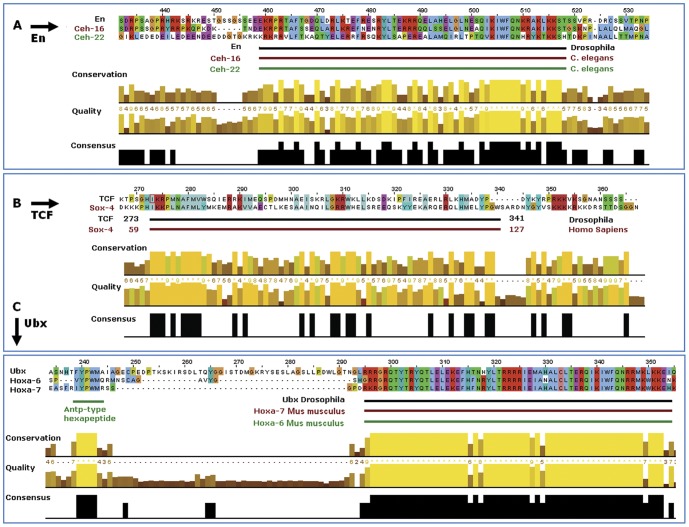
Results of the UniPROBE search using putative TFBSs for three TFs as a query. The search allows checking homology to known binding sites from different species. The results of sequence alignment are shown for three TFs: En (Panel A), TCF (Panel B) and Ubx (Panel C) with most similar binding proteins reported from UniPROBE. For Ubx we found ortholog Hoxa-6, which shows perfect similarity with Hox-7 reported by UniPROBE.

The three panels in Figure 2 present the three alignments among the amino acid sequences of the best matches and target proteins from Table 2. For comparison, we added the known homologs Ceh-16 and Hoxa-7 also found in UniProt. The bars at the bottom of the aligned sequences are placed according to the amino acid coordinates of the binding domains published in UniProt. The numbers at the top of each panel show the amino acid coordinates of the first protein from the proteins submitted to ClustalW2. Three conservation histograms (feature of JalView) in Figure 2 show a high similarity among the aligned protein sequences including the targets En, TCF and Ubx. In particular, from the conservation histogram in panel A, more than 50% of positions showed at least 70% sequence identity for En, Ceh-16 and Ceh-22; similar results are shown in panels B and C. These alignments suggest that Hoxa-6, CEH-22 and Sox-4 have similar binding domains and therefore are likely to have similar DNA-binding preferences, providing support for the UniPROBE analysis.

**Figure 3:**
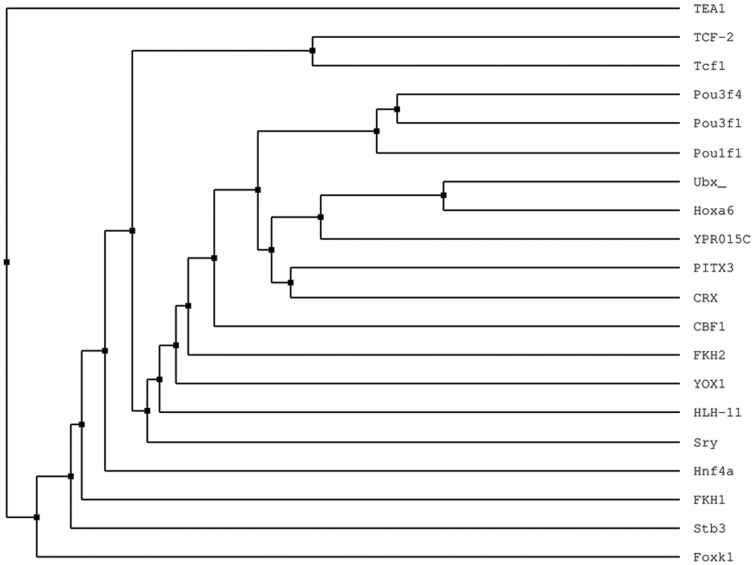
The tree shows variability of putative sites discovered using optimized dinucleotide matrix of UBX TF. Entry for UBX was not present in UniPROBE at the time of analysis, however, proteins Hoxa-7 and YPR015c mapped to queried putative TFBSs for UBX with top E-values show closest distance to UBX. Hoxa-7, which has UniProt entry Hoxa-6, *Mus musculus*, is reported as the ortholog of UBX in *Drosophila*. Proximate proteins showed unbiased distances to UBX. Notably, UBX was placed in the middle of the tree by the unsupervised method suggesting that our approach is unbiased.

The smaller length (7 nt) and the bigger number of queried PWM-sites for Ubx (than for En and TCF) are consistent with the bigger number of UniPROBE matches. Although we did not quantify dependencies between the number of UniPROBE matches and their quality, we visualized the result of the alignment as a rooted UPGMA (Unweighted Pair Group Method with Arithmetic Mean) tree constructed based on the percentage of identity, as computed by JalView (Figure 3). For this analysis, we used proteins of a length similar to Ubx and excluded proteins marked “non-characterized” or “predicted”; Ubx sequence was included as a reference. The tree confirmed the E-value result for Ubx from the UniPROBE report in Table 2, in which Hoxa-6 is closely related to Ubx based on the amino acid sequence alignment.

The analysis above indicates that TFs identified by UniPROBE as having similar DNA binding preferences to TFs of interest also had similar amino acid sequences. Based on this similarity, we reasoned that these TFs may also share similar biological functions. To investigate the biological roles of TFs identified by UniPROBE, the TFs used in sequence alignment and their homologs, we conducted literature reviews.

Beyond the sequence alignment which indicated closely overlapping protein binding domains in Figure 2, we could not find experimentally verified homologs between TCF and Sox-4, although work by Van de Wetering *et al*. mentioned that TCF-1 and Sox-4 are highly homologous factors and members of the same protein family [19].

Another TF, Ceh-16 was reported to be an ortholog of Engrailed (information is available at http://www.genecards.org/cgi-bin/carddisp.pl?gene=EN1&search=ceh-16); Hoxa-7 (Mm) was reported as homolog of Ubx (information is available at http://www.genecards.org/cgi-bin/carddisp.pl?gene=HOXA7). Three proteins, Ceh-16, Ceh-22 and En, have homeobox DNA binding domains as annotated in UniProt. Hoxa-6 (from UniPROBE) and Hoxa-7 (was not in UniPROBE) are members of the Antp homeobox family and also have a co-localized DNA-binding domain, which was confirmed from our analysis as shown in Figure 2.

During this study we checked if Match™ would be able to identify the same TFs as UniPROBE reported on our queries for the PWM-sites. To address this question, the same kind of analysis was conducted for Ubx, En and TCF using putative binding sites predicted by Match™. We again queried UniPROBE with Match™ predicted sites using the UniPROBE search tool to find corresponding TFs. Although in both cases close homologs were found (Figure 2), all PWM-sites for optimized PWM resulted in a much higher number of hits and smaller E-values to similar motifs (from UniPROBE collection of TFs) than sequences discovered by Match™. E-values of Match™ as indicated in the summary Table 3, are actually very poor.

**Table 3:** Summary of analysis Match™ vs. optimized PWM vs. UniPROBE search for similar motifs.

Sequences found for TF	Match™ num. of hits	Match™ best E-val.	OPT PWM num. of hits	OPT PWM best E-val.	Matched with
En	1	0.0429540	12	0.000305	CEH-22
Ubx	1	0.361303	7	0.003571	Hoxa-6
TCF Q6	2	0.107297	5	0.008115	Sox-4

We then further assessed the biological relevance of the new predicted TFBSs. To do so, we performed a mutual comparative analysis of DNA motifs and amino acid sequences using a collection of DNA binding motifs from high resolution protein binding microarrays. This kind of analysis is limited (therefore potentially biased) by available information found either in databases or in publications. Nevertheless, for three TFs we were able to find evidence from different sources of information that at least some of the new putative PWM-sites carried binding properties discovered in homologous or even orthologous TFs. These findings were obtained from querying the UniPROBE database by new putative TFBSs and confirmed by aforementioned publications. For this reason we can consider that the refined PWMs are reasonable as tool for computational prediction of new TFBSs. In remaining section, we assess the quality of refined PWMs as predictors using mostly quantitative comparative analysis with Match™.

Novel sequence content discovered by optimized matrices is consistent with what was biologically proven for binding affinity. To illustrate this statement we considered the information available for transcription factor Zeste (Z). Mutagenesis studies determined that (T = C = g)GAGTG(A = G = c) is the consensus Zeste recognition sequence [20]. Similarly, our results partly confirm this consensus based on both types of the matrices, as we found nine sequences (out of 92 using the optimized mononucleotide matrix) on the set of proximal promoters with clear (T = C)GAGCG consensus, which differs only by one nucleotide C from motifs in published study [20]. Consistently, the optimal dinucleotide matrix gives additional seven sites (out of 31 discovered) supporting the motif with C. Our observations are supported by two publications [21], [22] which found similar sites with GAGCG motif among the experimentally characterized binding sites. Hence, the optimized PWMs are able to bring new insight on binding specificity of TFs that might be consistent with those examined in literature. The novel binding sites we found complement four TRANSFAC sequences (among 24 original) that we used for training. The list of sites mentioned in this paragraph can be found in Table S2 [in File S1].

Providing two types of optimized PWM in this study we are not engaging them in a contest. In turn, the final example shows that using both types cooperatively is able to shed an extra light on a research.

Using a mononucleotide optimized matrix, we detected En putative binding sites upstream of the TSS in a number of promoters. Computational discovery of these sites using PWM is a challenging task because at least three nucleotides in the consensus En binding motif coincide with those in the following overlapping sequence TCAGT, also known to be one of the overrepresented sequences in *Drosophila* genome, mostly at Initiator (Inr) sites [23]. Nevertheless, both optimized matrices recognized noisy matching signal from En TFBS upstream of the Inr site, in agreement with prior work [24]. Comparing dinucleotide with mononucleotide PWM versions, one may see that “G” is eliminated in putative binding sites obtained by the dinucleotide matrix. Figure 1 shows the routine dependency analysis between mono- and dinucleotide occurrence frequencies for En compared with Zeste TF. This figure shows that adjacent nucleotide positions are mostly dependent and hence the dinucleotide matrix predicts such motifs better, in agreement with experimentally obtained sequences.

### Optimized PWMs show an increased accuracy in simulations

The sequence Logos obtained in Figure 1 revealed that we could find new putative PWM-sites that might be relevant to TF-DNA binding. However, this analysis did not provide us with a comprehensive examination of predictive performance of the new refined matrices. To fill this gap we constructed tests on synthetic sequences, a popular *de facto* instrument for testing of new computational methods [16].

In the Introduction we discussed the Match™ software, supplied with TRANSFAC commercial database and used with its original PWMs. Predictions from the application of mono- and dinucleotide optimized matrices (further indicated with the prefix “OPT”) were compared separately with generic mononucleotide matrices used in Match™. The goal of such a testing was to compare the number of sites predicted by each method at expected locations. A summary of all test results is shown in Tables 4 and 5. We split results into two tables because the Match™ distinguishes the high and low quality matrices presented in Table 4 and Table 5, respectively.

**Table 4:** A summary of experiments with synthetic data tests for high quality TRANSFAC™ matrices.

Name	Measures	Match™	Match™	Match™	OPTmatr	& opt. cut-off	Test size
		min FP	min FN	both crit.	mono	di	
KR	TP	2	21	12	15	11	31
	FP	0	19	2	3	0	
	Precision	1	0.525	0.857	0.833	1	
	Recall	0.065	0.677	0.387	0.484	0.355	
	CC			0.575	0.624	0.595	
SuH	TP	0	10	6	10	10	10
	FP	0	1	1	1	1	
	Precision	n/a	0.91	0.857	0.91	0.91	
	Recall	0	1	0.6	1	1	
	CC			0.716	0.953	0.953	
Z	TP	6	21	20	14	14	41
	FP	1	144	82	17	2	
	Precision	0.857	0.127	0.196	0.452	0.875	
	Recall	0.146	0.512	0.488	0.341	0.341	
	CC			0.305	0.391	0.529	
E74A	TP	3	16	9	11	0	17
	FP	1	38	6	1	0	
	Precision	0.75	0.296	0.6	0.917	n/a	
	Recall	0.17	0.941	0.529	0.647	n/a	
	CC			0.562	0.77	n/a	

The results for Match™ and optimized matrices (OPT) are organized in blocks. Match™ consists of three tested types of settings: “min FP” - minimum number of false positives; “min FN” - minimum number of false negatives; “both crit.” - min of sum of both criteria; “m” and “di” - refer to the number of hits after the application of mono- and dinucleotide matrices, respectively. TP rows show the number of retrieved TP hits (out of number showed in test size). Cut-off values for optimized matrices were selected as they appeared after optimization.

**Table 5:** A summary of experiments with synthetic data tests for low quality TRANSFAC™ matrices.

Name	Measures	Match™	Match™	Match™	OPTmatr	& opt. cut-off	Test size
		min FP	min FN	both crit.	mono	di	
En	TP	0	5	5	2	3	12
	FP	1	21	9	60	1	
	Precision	0	0.1923	0.3571	0.0323	0.75	
	Recall	0	0.4167	0.4167	0.1667	0.25	
	CC	0	0.2796	0.3835	0.0663	0.432	
Abd-A	TP	0	7	2	4	8	23
	FP	0	59	12	36	45	
	Precision	n/a	0.106	0.143	0.1	0.151	
	Recall	0	0.304	0.087	0.174	0.348	
	CC	n/a	0.175	0.109	0.128	0.225	
PRD	TP	0	13	0	2	1	37
	FP	3	183	12	27	5	
	Precision	0	0.0663	0	0.069	0.167	
	Recall	0	0.3513	0	0.0540	0.027	
	CC	0	0.0754	0	0.0582	0.0659	
Ubx	TP	0	3	1	15	15	20
	FP	2	77	27	294	25	
	Precision	0	0.038	0.036	0.049	0.375	
	Recall	0	0.15	0.05	0.75	0.75	
	CC	0	0.0688	0.0384	0.1826	0.5282	
Sna	TP	0	12	2	0	3	40
	FP	2	214	15	0	7	
	Precision	0	0.053	0.118	n/a	0.3	
	Recall	0	0.3	0.05	n/a	0.075	
	CC	0	0.0119	0.0746	n/a	0.1486	
KNI	TP	1	18	14	4	0	26
	FP	0	80	16	4	0	
	Precision	1	0.184	0.467	0.5	n/a	
	Recall	0.038	0.692	0.538	0.154	n/a	
	CC	0.1958	0.3528	0.4994	0.276	n/a	
DEAF1	TP	0	4	1	2	2	10
	FP	0	11	4	7	7	
	Precision	n/a	0.267	0.2	0.222	0.222	
	Recall	n/a	0.4	0.1	0.222	0.222	
	CC	n/a	0.3238	0.1394	0.2083	0.2083	
AP Q6	TP	0	0	2	0	0	20
	FP	1	133	26	41	34	
	Precision	0	0	0.071	0	0	
	Recall	0	0	0.1	0	0	
	CC	0	0	0.0714	0	0	

The rows and columns specified as in Table 2.

As was mentioned, all synthetic test data were constructed from the JASPAR collection of sequences. The JASPAR collection of TFs is smaller than that of TRANSFAC. Match™ and OPT PWMs were applied in parallel for each of these synthetic samples and the results are summarized in Tables 4 and 5.

Since Match™ proposed three distinct types of the cut-off profiles optimized for different criteria we performed tests for each. The rationale for these three cut-off values was three-fold: 1) to minimize the number of biologically relevant binding sites missed by Match™ (min FN), 2) to minimize the number of random matches found (min FP), and 3) to minimize these combined error rates (min (FN+TP)). To address these three search criteria we used precompiled profiles supplied with Match™ and extended synthetic test sequences to the equal length of 300 nt. At this stage TLL TF was excluded because of an absence of TLL profiles in Match™ collection. We also excluded Hb TF because in presented JASPAR collection all sequences were redundant with TRANSFAC Hb sequences that we already used for matrix training process.

**Figure 4:**
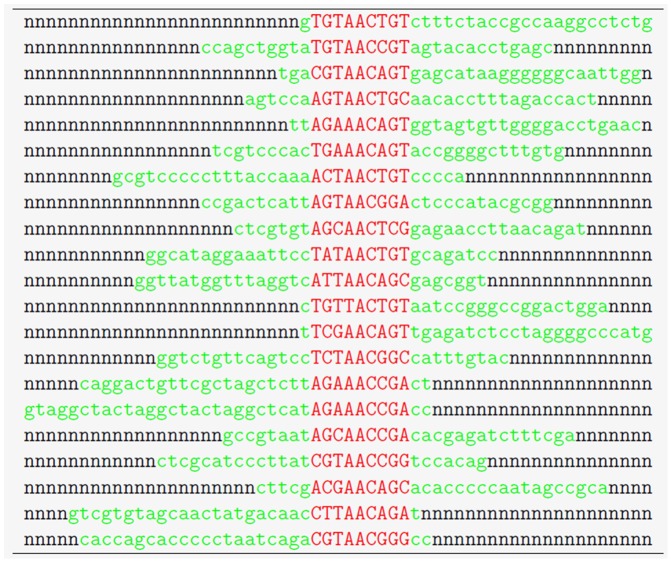
A fragment of the synthetic data test example constructed from JASPAR sequences. Alignment of JASPAR sites is highlighted in red and start of the alignment is centered at a fixed known position throughout the current promoter set at position 27 in this example. Equally distributed random nucleotides “*n*” extend each promoter sequence to achieve equal length.

The impact of the sequence background was reduced by taking ten replicates, each of which obtained by the random reshuffling (30 times) of the nucleotide background surrounded the inserted flanks. Unaligned parts were preserved in replicates, although we were able to permute them at each position (“green” nucleotides in Figure 4). To include possible shifted matches, we enlarged the search area in favor of Match™ during hit counts. This means that if Match™ detected a hit within a half of the motif length ±1 nt from closest motif edge we considered this hit as correct. The focus of our experiments with synthetic tests was to examine which matrices predict TFBS locations better – the OPT PWMs or generic TRANSFAC matrices which Match™ uses. Unlike OPT PWMs, Match™ locates the hit position at the first position of five consecutive the most conserved positions within the matrix. This key difference might cause a shift which was accounted during the hit count.

It was a challenging goal itself to compare the results of low quality PWM because we could not be certain about an origin of their low quality classified by Match™. For the same reason we cannot suggest that those matrices became good or not now, however, our analysis showed that the total averaged predictive performance of OPT PWMs significantly improved. Another extra challenge was to find the statistics to measure the predictive performance when we expected overwhelming or missing data for low quality matrices.

To integrate differences in predictions of two methods on synthetic tests we computed indexes described in Method part, which also includes Mathews correlation coefficient which consolidated TP, FP, true negatives (TN) and false negatives (FN) from each test in one index of predictive quality of OPT and Match™ methods. Table 4 and Table 5 also include characteristics complementary to precision and known as recall (sensitivity).

As seen from Table 4 for high quality PWMs, the optimization results in at least equal or better characteristics of predictability than Match™ method based on generic TRANSFAC high quality matrices. We compared the hits found by the optimized matrices with optimized cut-offs on the synthetic tests with the hits obtained by Match™ with its accustomed profiles. Noticeably, both mono- and dinucleotide optimized matrices give better results comparing to Match.

Table 5 contains the result for low quality PWMs. We were unable to break through the low performance of AP Q6 matrix and produce any improvements. KNI matrix tests showed a better performance for Match™ method. It was not surprising at all because as we found the length of optimized matrix for this TF was much longer than length of sequences presented in JASPAR and this result means that we should exclude this TF from analysis.

Unlike the results for high quality matrices, the mononucleotide matrix in Table 5 was not undoubtedly better than Match™, but dinucleotide matrices still remain better almost through all tests. Noticeable to mention about an ambiguity in counting hits from Match™ results. Unlike the high quality matrices, for low quality matrices it was not easy to identify the systematic shift (originated from the Match™ detection method) directly from the hits report. Although this shift associated with a certain position within PWM and should be invariant of selected PWM (by Match™) we are not able to estimate the shift without direct computations.

To resolve this situation we accounted all positive hits within matrix length in favour of Match™. As a result, Table 5 collected a bigger number of TPs than it actually should do. Even so, from Table 5 we see that in all cases Matthews correlation coefficient gives bigger values for all optimized dinucleotide matrices and for almost all optimized mononucleotide (with exception of En) when they compared to results of Match™ with its cut-off profiles accustomed for minimum of TPs and FPs. The same comes from the precision characteristics. JASPAR testing collection for En has seven overlapping sequences with sequences from TRANSFAC and this is why the Match discovered them easily.

Nevertheless, the generalized pattern of predictability is better for OPT matrices. We performed unpaired right-tailed t-test for available Matthews correlation values from Tables 4 and 5, and found that they are significantly bigger for OPT PWM predictions (with 90% and 95% confidence values for mono- and dinucleotide respectively).

The conclusion from this analysis is that optimized PWMs work better on new promoter sequences. In addition, based on the structure of our synthetic tests used we can assume that OPT PWMs are a better choice for *in silico* TFBS prediction than Match™, in mapping of TF binding positions.

## Discussion

Results of our computational experiments show that optimized matrices can successfully detect binding sites on a test data set constructed independently of the training data sets. This demonstrates that our machine learning approach resulted in PWMs with better predictive performance than generic TRANSFAC matrices.

Another insight from our results is the demonstration that our adjusted heuristic framework makes the optimization approach implementable in a large scale for TRANSFAC TFBSs collection for *Drosophila* and it results in a better global performance compared to the conventional approach [25], [26].

The further development of the refinement technique was a logical continuation of the previous work of Gershenzon *et al*. which implemented the core idea for the PWM of the GC-box binding site for Sp1 TF [4]. Its application to a larger scale was, however, limited by the manual inspection of promoter area for the presence of signal and demanded converged optimization process. As a result, this heuristic procedure was applicable only for a limited number of suitable PWMs. In our improved algorithm, which included the biological signal detection part and controlled steps over all optimization process, we provided a set of heuristic rules combined into one stable and recyclable model. Optimized matrices were used to predict up to a thousand new putative binding site candidates in the SIB-EPD set of promoter DNA sequences for *Drosophila* (as shown in Table 1, columns 6 and 7). The resulted method described here involves the training of PWM on novel biological sequences which extend a set of available TFBSs by using statistical similarity. Unlike in sequence alignment, which is central to comparative studies, the similarity we are exploiting in the algorithm comes from the possible statistical variability of positionally dependent nucleotides in predicted proximal areas of promoters (in detected functional window).

It is worth mentioning that the number of sequences predicted by optimized dinucleotide matrices is roughly half the number of sequences predicted by optimized mononucleotide matrices (two last columns in Table 1). In terms of nucleotide counts, it means that the optimized mononucleotide matrices gain as many as twice more nucleotides per TF compared to nucleotides in sequences discovered by the dinucleotide matrices. In contrast, the count of nucleotides in the initial training sequences (“S.Used∶L” column in Table 1) was ten times less than that after the application of optimized mononucleotide matrices with an optimal cut-off. Since we used the same functional windows in both matrices, this phenomenon illustrates that mono- and dinucleotide matrices complement each other in a way that the mononucleotide matrix aims to extend the diversity of nucleotide content, whereas the dinucleotide matrix works to preserve biological relationship between neighboring positions and thus both goals are achieved by using both matrices. As a result, dinucleotide matrices predict sequences that look more similar to the original ones (compare Logo's pictures (B to A) vs. (C to A) in Figure 1). The entropy measure expressed in sequence Logos supports the interpretation of that similarity.

The method used to construct optimized matrices confirmed its superior combination of sensitivity and specificity compared to the conventional approaches. Better specificity compared to the conventional approaches was reported as a feature of optimized matrices in earlier publications [4], [13] for single TFs. Here we extend this observation to batch processing of TFs. Low specificity is a common limitation of conventional TFBS prediction algorithms, and therefore the improvement in the specificity (while maintaining a level of sensitivity) of our method is a significant accomplishment.

From the inspection of discovered sequences, we note that putative binding sites contain variable nucleotide contents at certain positions as a result of more or less significant degeneracy in binding motifs at that position, which can bring additional insight into the binding affinity of certain TFs. For transcription factor Z (Zeste), for example, nucleotides next to and immediately after the GAG pattern are interchangeable (T or C), although the occurrence of pyrimidines at those positions was confirmed [20].

Figure 1 shows for Ubx that the position after the first T in TAAT is more degenerate than the remaining nucleotides. For the Ubx TFs, we predicted sequences with ATTA and TAAT motifs which were reported as a preferential combination for Ubx homeodomain protein [27], [28], [29], [30] when it binds *in vitro* in a sequence-specific way. This type of motifs is observed in both dinucleotide and mononucleotide (although shortened) sets of PWM-sites, which advocates in favor of their biological relevancy. In this example we capture TAAT and ATTA patterns in the discovered sites. However, for some reasons they were missed in the TRANSFAC although we detected such sequences with refined mononucleotide PWM.

Intuitively, one can expect that a hypothetical method capable of finding more similarities to the initial binding sequences would perform more favorably compared to a method that does not find such similarities. This statement, for example, might be affected by stochastic irregularity of promoter content, by completeness of TFBSs collection used for training and their biological degeneracy (positional variability), and thus, in fact, the declaration is not true in all cases.

Z-score is used as a tool to detect a promoter area with significant peak matching scores. Conceptually, the z-score used for hit counts is similar to the averaged positional distribution of elements along the aligned promoter sequences published [4], where the expected occurrence frequency is taken for a randomized (shuffled) promoter content. Our experiments with z-scores pointed to the same promoter areas, though indicating smoother distribution with fewer and more expressed peaks that facilitate large scale implementation. The number of available TFs would have increased to 64 TRANSFAC entries at the time of the study if we also had included the data where only mononucleotide PFM frequency tables were present. Thus, in our study the presence of TFBSs in the database was a limiting factor in selection of TFs.

We complemented mononucleotide matrices with dinucleotide versions of PWMs for the following two reasons:

- a dinucleotide matrix indicates dependencies between adjacent nucleotides and infers binding scores from commonly used thermodynamic model that minimizes binding energy of protein DNA-binding motif;
- a dinucleotide and mononucleotide matrices can be similarly computed. The pairwise computation allows the assessment of the computational schema capability to discover similar putative sites implying mutually complementary concepts: assuming or discarding the Markovian property between neighboring nucleotides. An answer was not obvious from the onset of our analysis; in particular, those similar solutions could be found for all matrices of interest using both concepts especially when considering irregular nucleotide background distributions near TSS. We found more or less similar patterns for all 33 TFs considered where biologically verified sequences were available.

Binding sequences used for training are limited in quantity (Table 1, column 4) for most TFs used. Therefore, one may not expect good sampling while performing matrix training because good sampling presumes confident knowledge of the whole range of binding specificities. Surprisingly enough, in all cases we were able to reasonably extend the number of sites which show matches with used proximal promoter source. As an example, for dinucleotide matrices, we found new putative sites that are in good agreement with experimentally confirmed patterns. This is illustrated by the observation of two most abundant nucleotides at each position. Moreover, the dinucleotide patterns are more often similar to those derived from the initial sequences. This provides evidence that additional information brought from dinucleotide pairs is able to compensate for the scarce information if only a few binding entries are present in the data. Consistent with this is the observation that the top of the Logo pattern of the dinucleotide version is more conserved compared to the initial Logo pattern. On the other hand, mononucleotide versions often show more variability at certain positions. Examples of a lesser variability: for TF Abd-B strong nucleotide T at position 3; for TF Ubx - G at position 4 (Figure 1); for TF KRQ6 - C at position 5 (see KR Logo Figures from File S2). (The numbers correspond to the nucleotide order in the initial TRANSFAC sequences).

We used data from TRANSFAC commercial database for training purpose and JASPAR binding sequence entries for testing. Although the JASPAR public data collection is not as large as the commercial TRANSFAC collection, the former database delivers smaller numbers of manually curated sites suitable for testing. In many cases (for example En, Ubx, BRK) we found discrepancies between the JASPAR and the TRANSFAC sets, as seen in the sequence Logos, which was the source of an extra challenge for the optimized matrices to predict sequences listed in JASPAR in such cases, as our optimized matrices are based on the TRANSFAC training data. Tompa *et al*. argued that type of promoter content greatly impact TFBS predictions [16]. Using their classification of promoter models as “real”, “generic” and “Markov”, we can present our result as follows. Optimized matrices, trained on one set of binding sequences with “real” promoter content, were able to identify biologically relevant sequences on an alternative “generic” promoter content. Whereas composition of fair and effective tests is an extra challenge for computational biologists, the capability of optimized matrices was significant for all refined matrices from TRANFAC for which we were able to perform independent testing.

Quantifying the actual impact on investigating transcription regulation, such as demonstrating improvement gained from the new models on both genome wide information, as well as enhancers in specific regulatory networks, would be a possible future direction of the PWM refinement proposed in this study.

## Materials and Methods

### Materials

The quality of the computational predictions of new TFBSs builds upon the quality and quantity of available binding specificity information. As in the previous work [4], we used the Eukaryotic Promoter Database (EPD) (release 105) as source of promoter sequences (available at http://epd.vital-it.ch). Along with EPD, to compute a collection of the PWMs for *Drosophila* TRANSFAC database (release 2009.4) was used (available at http://www.gene-regulation.com).

To enable a compilation of both mono- and dinucleotide PWM types, we utilized 33 TRANSFAC TF entries for *Drosophila* for which binding sequences were presented. In addition to EPD and TRANSFAC used for training of refined PWMs, JASPAR database was used for PWM testing (available at http://jaspar.cgb.ki.se/). We occasionally used also other data sources for the PWM testing and for characterization of few discovered TFBSs.

JASPAR database contains a manually curated, mostly non-redundant with TRANSFAC sets of profiles, derived from published collections of experimentally defined TFBSs for eukaryotes [12]. The JASPAR collection provided us with an extra challenge when used for PWM testing of predictability because most of the sequences in JASPAR are longer than just binding motifs.

Due to differences between sequence contents of TRANSFAC and JASPAR, the former was mapped against JASPAR's core collection. For each of the 33 TF entries (by matrix accession codes) from TRANSFAC we tested the corresponding TF from JASPAR core collection. Rare cases of ambiguity in names were resolved using NCBI official gene names and their aliases. We found 14 TFs in common and used them to construct our testing sets, as described in the next subsection.

Match™ tool of the TRANSFAC was used for benchmarking the optimized PWMs. An additional benefit of using Match™ was a comprehensive access to whole capacity library of mononucleotide matrices provided with commercial subscription of TRANSFAC.

The EPD database was employed as a source of biologically confirmed information for searching for new viable binding motif sequences. The EPD promoter sequences for *Drosophila*, each 600 bp long (−499 to +100 bp) aligned and centered on the position of a TSS, were used in two ways: (a) as a target set to mine new putative TFBSs (these sequences were scanned during PWM refinement), (b) EPD sequences were reshuffled to simulate randomized background sequences used in PWM calculation [4]. Promoter sequences containing unknown nucleotides (denoted as “*n*” or “*N*”) were excluded from these computational experiments and remaining 1919 sequences were used. The length of proximal to TSSs areas was selected to be able to include potential locations of all target TFs (binding primarily in a proximal promoter area) selected for this study. Enhancers situated in much broader regions of a distal promoter area were not a subject of the present study.

#### A structure of synthetic tests from JASPAR data

A synthetic test set was constructed for each TF (14 in total) used in a JASPAR testing set. Each JASPAR sequence candidate for this testing was assessed for its non-redundancy against the corresponding TRANSFAC sequences. We preserved original sequences presented in JASPAR with a variable length for each tested TF. The original JASPAR local alignment of the TFBS sequences (marked red in Figure 4) was also preserved. Then, we located all aligned parts at certain position (for example, position 27 in Figure 4) and embedded the aligned and non-trimmed sequences into a random sequence environment. The resulted synthetic test structure is shown in Figure 4 where “n” indicates random nucleotide content.

The known location of TFBSs facilitates the count of true and false TFBS matches during PWM tests.

### Methods

We exploit an idea [2] that motifs necessary for transcriptional *cis*-regulation should be overrepresented in a particular area of promoter region for a variety of TFs. We call a particular binding site over-represented in some promoter area if it occurs there more frequently than in a respective randomized sequence under conditions of certain statistical model. Abundance or rarity assessment of TFBS *m* comprising *L* nucleotides can be estimated by comparing it's observed to that expected occurrence frequency in a certain promoter area. While it is generally accepted that the overrepresentation of specific oligonucleotides is a statistical aspect of DNA sequences with biological relevancy, ways of quantifying overrepresentation are quite diverse and vary from exhaustive direct search to numerical filtering. Unlike exhaustive sequence-based enumeration [31], PWM approach to counting frequencies involves calculating *z*-score for a number of sequence *S* occurrences (hits) in that promoter area by applying initial PWM *W* with certain matching cut-off *c*, (all together summarized as a suite model *<W,c>*) in a window of length *L* gradually sliding downstream the aligned promoter sequences:
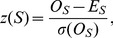
(1)where σ and *E* are symbols of standard deviation and expectation of the positional hit count, respectively. Z-score is a measure of standardized difference: observed *O_S_* minus expected *E_S_* over the standard deviation σ(*O_S_*). The expected frequency is estimated as the mean of all hits over all positions in the promoter set divided by the numbers of these positions and promoters.

Z-score is utilized in a variety of ways in the genome research. It was recently used to select gene–targets for a particular TF using PWM and gene expression data [32]. Similarly, we use it here as to narrow potential location of putative binding motifs to promoter segment with overrepresented hits which can indicate presence of majority of functional binding sites and is interpreted as a “functional window” [4]. For this purpose we selected areas with maximum z-score values 3 and more computed for the initial mononucleotide PWM.

Thus Equation 1 for *z*-score was used for search of overrepresented binding motifs. Overrepresentation is defined by excessive number of hits in promoters versus those in randomized sequences when PWM with certain selected cutoff is applied. The *z*-score is calculated at each sliding window position using mononucleotide matrix [4] from source TFBS sequences (which can be computed from an occurrence frequency table). It shows an averaged positional distribution of oligonucleotides along the promoter sequences aligned according to TSS position. That positional distribution used to estimate a functional window on scanned promoter sequences.

Weight 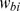 of nucleotide *b* at the *i*-th position in a sequence motif is a component of mononucleotide *4×L* - dimensional matrix of length *L* calculated as corrected log-odds ratio [4]:
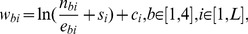
(2)where
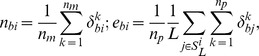

*s_i_* in parentheses is equal to zero, if 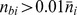; it is equal to 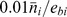 otherwise.


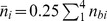
 is the expected fraction of bases at position *i*. Here *b* is an index of a nucleotide from ordered set (*A*,*T*,*G*,*C*), *n_bi_* is the average number of times base *b* occurs at the *i*-th position of motif, *e_bi_* is the expected frequency of *b* base at position *i*. Limits in sums *n_m_* and *n_p_* are numbers of binding sites in training set and number of promoters respectively. For k-th promoter, sequence symbol *δ^k^_bi_* organizes a counter by index *k*. *δ_bi_* equals to one, if nucleotide *b* occurs at the position *i* and equals to zero otherwise. Value 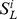 describes sliding area of sequence of length *L* which begins at the current position *i* and ends at the position *i*+*L*−1. To calculate *e_bi_* we took the promoter area from −500 to +100 nt, so *n_p_* taken instead of *n_m_*; 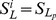 and *e_b_* does not depend on a sliding position *i*. This helps us with a simpler notation as well, as we applied in dinucleotide version (Equation 4). We adopted this PWM model after [4] because it accounts for non-uniform nucleotide background distribution that improves TFBS prediction. This is important when only small number of binding sites is available to compute PWM. Position-dependent constants *s_i_* and *c_i_* are chosen to support positive area of logarithm and to scale the maximum individual nucleotide score value in each i-column at zero, respectively. Values *s_i_* are responsible for position weights of very rare nucleotides. In our PWM refinement algorithm, instead of applying similarity between an oligonucleotide and PWM [33, Formula 2] or rescaling PWM weights [4, Equation 2], we implemented matching scores S(seq) directly to weights as shown in Equations 3 and 5. This means that the matching score *S(seq)* for specified sequence motif 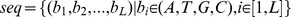 is computed as:
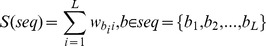
(3)


Thus, Equation 3 is similar to the one used in [4] but does not use normalization.

The version for dinucleotide PWM is shown below (Equation 5) and implemented the same principle. Both Equations are used as scanning tools to compute and process matching scores in consecutive sliding windows on promoters. Details of the algorithm are described in the following section.

Weight of *d*-th dinucleotide at *i*-th position *w_di_* in a sequence motif according to [4] is a component of 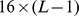-dimensional matrix, which can be formalized in the following way:
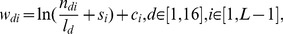
(4)where
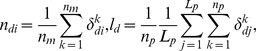

*s_i_* is equal to zero if 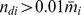; it is equal to 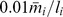 otherwise. More specifically, dinucleotide matrix *w_di_* is composed from 16 rows, each row corresponding to a di-nucleotide in the following order: AA, AT, AG, AC, TA, TT, TG, TC, GA, GT, GG, GC, CA, CT, CG and CC. Parameters *n_di_*, *l_d_*, *s_i_*, *δ^k^_di_* and *c_i_* liken corresponding parameters from mononucleotide case, but taken for dinucleotide sequence and 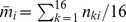.

For dinucleotide PWM the matching score *S* of specified sequence *seq* calculated as weight score of derived di-nucleotide sequence *dseq*, where
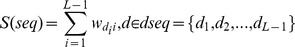
(5)Matthews correlation coefficient *Cor* has been used in the optimization procedure as it was done in our earlier publication [4]:
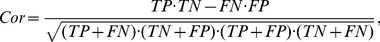
(6)where TP, FP, TN and FN are the number of true positives, false positives, true negatives and false positives respectively. Matthews coefficient is a convenient form to combine sensitivity and specificity in one equation [15]. We address a reader to the Algorithm part which describes applications in more details.

For comparison of our results with other results from synthetic tests we used the positive predictive value (also known as precision) - index easily computed as a ratio of TP over test outcome positives: precision = TP/(TP+FP). A complementary characteristic is the sensitivity (also known as recall): sensitivity = TP/(TP+FN). In analysis of PWM applications the definition of components of confusion matrix is the following: TP – is the number hit positions (one per each test sequence) which coincide with location of upstream first nucleotide in aligned JASPAR sequence or shifted within PWM length; FN - number of sequences from the synthetic test that remained undiscovered (without hits pointed on them); FP – number of incorrect hits; TN - number of all possible negative matches for the selected test size, which is the complement of sum (TP+FP+FN) to the total number of possible hits in the test.

### Algorithm

An assessment of the abundance or rarity of *L-*nucleotide long TFBS can be estimated by comparing its observed frequency to that expected in a certain area on promoters. Unlike exhaustive sequence-based enumeration [31], our PWM approach to counting frequencies involves calculating z-scores for a number of sequence occurrences (hits) in that promoter area by applying the initial PWM ***W*** with certain matching cut-off *c*, together summarized as a suite model *<* ***W*** *,c>*. This search involves several biological contents including the matrix specificity model and localization on promoters.

The implementation of the model for a large-scale application consists of the following three stages. Second and third stages are iteratively repeated until a final solution is found.

1. Selection of binding sites and preprocessing: inspection of a representative list of TFBSs avoiding gaps and sequence replicates. If necessary, the preprocessing includes sequence alignment and truncation to a fixed length. The final stage of preprocessing is compiling the initial PWM which accounts for the background nucleotide distribution.
2. Biological signal detection: preliminary scanning of promoter sequences to find potential areas of new TFBSs. This stage estimates the initial cut-off; cut-off boundary values and the functional window (or windows).
3. Finding similar motifs in promoter sequences. This is an iterative processing for similar motifs in the functional window, using the range of PWM cut-offs; compiling the refined PWMs from discovered auxiliary new sites; optimizing the functional window, motif length, and cut-off. Matthews correlation coefficient *Cor* (Equation 6) increases monotonically until it reaches the maximum of 1 or when increase terminates (whichever comes first).

The first and second stages are the preliminary stages followed by the third, core stage of the iterative matrix refinement process. The automated second and third stages are implemented in the original core MATLAB/Octave code not invoking any specialized toolboxes. A Perl script computes the PWM matrix using BioPerl package for sequence handling.

#### Selection of binding sites and preprocessing

Since our software is designed to evaluate equally sized binding sites without gaps, preprocessing consists of mostly manual selection of binding sequences from the database. We preserved existing TFBSs alignments if they were published in the database. However, in cases of E74A, Ubx, Dl (Dorsal), MtTFA, DREF, the TFBSs were listed with different length so we performed our own alignments. For some TFs such as Ubx, available in TRANSFAC with 88 sites, we computed matrices from distinct common aligned parts trying to preserve most of the known binding specificity, but the remaining TFs were preprocessed as they were. The preprocessing stage ends with initial mononucleotide and dinucleotide matrices that are subjected to further training for optimized performance.

#### Biological signal detection

The PWM search often results in a large number of random matches. To limit the number of false positive TFBSs, we used an additional criterion, the z-score, in order to (a) locate a functional window and (b) estimate an initial PWM detection cut-off. We calculated z-scores (Equation 1) from number of hits detected above the cut-off based on Equation 3 for the mononucleotide case. The z-score was used as a measure of the overrepresentation of hits in a window of length *L* gradually sliding downstream the aligned promoter sequences.

**Figure 5:**
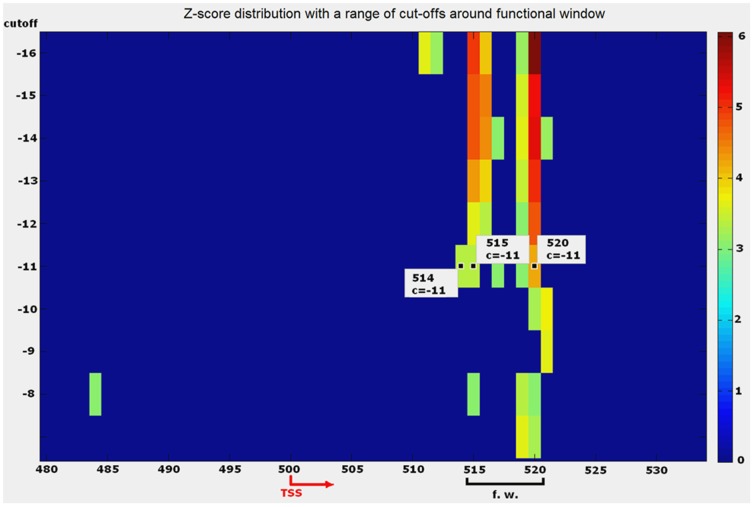
Z-score distribution on proximal promoter context of 1919 promoters produced by initial mononucleotide PWM. The colours show z-scores above three around TSS position of sliding windows for Su(H). Y-axe shows cut-off values ranged from most to less relaxed (top to bottom). Only a portion of proximal promoter area is shown. This graphical method demonstrates the way how initial PWM matrix cut-off *c* has been estimated.

An area of motif overrepresentation on TSS-centered promoters with qualified outliers has been estimated as an initial functional window. The length of the initial functional window is *L*, which is subject to further optimization. Since the z-score is sensitive to cut-off value, which is unknown *a priori*, we performed simple analysis of outlier distribution around the expected functional window with a range of consecutive cut-offs to elucidate the initial matrix cut-off and the most comprehensible functional window. The heatmap (Figure 5) shows an example for SuH.

There is no one-to-one unambiguous dependency between matrix cut-off values and qualified binding sites. While we expect that an accurate TFBS has a high z-score and is obtained under a stringent cut-off, in reality a more degenerate site can be identified with a less stringent cut-off [3]. A small number of accurate sites used to compute an initial PWM might also cause a statistical bias in score estimates and the resulting cut-off value. Another reason why it is impossible to establish one-to-one dependency between matrix cut-off and matching score is because PWM matching score is an additive composition of constant weights of individual nucleotides (Equation 3). In such circumstances, applying a spectrum of cut-off values helps to find the location of most sustainable signals. As seen from the example in Figure 5, the z-score at nucleotide positions 515–516 and 519–520, has the maximum magnitude, which is sustained up to a cut-off of −11. Because the distance between these two peaks is smaller than the motif length (which is 13 nt), stage 2 fuses these positions into a single widow.

Some authors have suggested the z-score as a measure of statistical significance to be used as an instrument to search for putative binding motifs [31], [34], [35]. Unlike z-scores used to detect motifs our approach simply uses z-scores to detect overrepresentation (outliers on a promoter area with highest z-score). An accurate location of a functional window is a big challenge due to two obstacles: a small number of TFBSs is available in the database and the short length of binding motifs may result in a high number of false positive hits. In a case when we detected more than one functional window we selected the window which gives the maximum correlation *Cor* with respect to the hits detected by the original PWM in this window and PWM after the first iteration.

#### Searching for similar motifs

The search for motifs is implemented as a completely automated routine. The method deals with mononucleotide and dinucleotide matrices alike and uses initial PWM ***W_0_*** within pre-estimated functional window and matrix cut-off. The goal of motif similarity search is to find new putative binding sites with maximum value of *Cor*. It starts from initial matrix/cut-off suite: ***<W^0^,c_0_>*** and passes through the following steps:

1. Apply *<* ***W^0^*** *,c_0_>* to search for new sites in a functional window.
2. Extend the initial set of sites with the new sites from step 1 and compute a new PWM matrix ***W*** *_i_*.
3. Apply matrix ***W^i^***. with cut-offs values *c* on the grid points ranging from *c_1_* to *c_2_* and find<***W^i^***, c_i_> with maximum *Cor* for initial combination *<* ***W^0^*** *,c_0_>*.
4. Use the new <***W^i^***,c_i_> to optimize motif length *L*. For this purpose, apply <***W^i^***,c_i_> and find a functional window for shorter and longer motifs applying *+/−* one nucleotide variation to original motif length. This step consists of four sub steps used sequentially, all within the same iteration step:
  - Use <***W^i^***,c_i_> to find hits and take corresponding sites truncated from left (abbreviated as “cl”) by one nucleotide. Compute ***W^i^***_cl_;
  - Use <***W^i^***,c_i_> to find hits and take corresponding sites truncated from right (abbreviated as “cr”) by one nucleotide. Compute ***W^i^***_cr_;
  - Use <***W^i^***,c_i_> to find hits and take corresponding sites extended to left (“el”) for one nucleotide. Compute ***W^i^***_el_;
  - Use <***W^i^***,c_i_> to find hits and take corresponding sites extended to right (“er”) for one nucleotide. Compute ***W^i^***_er_;
5. The resulting matrix (with the shorter or longer length) should be applied to a functional window using variable cut-offs valued from *c_1_* to *c_2_* to find the one *c* which yields maximum *Cor* with the previous pair <***W^i^***,c_i_>. Take this best as an initial matrix with cut-off value for the next optimization cycle, which repeats all the aforementioned steps beginning from step 1.
6. Reassign <***W^i^***,c_i_> as the new *<* ***W^0^*** *,c_0_>*, increment over i and repeat all aforementioned steps 1–5 until value *Cor* reaches the maximum or when increase terminates (whichever comes first).

During the procedure of matrix refinement TP, FP, TN and FN were redefined at each step. The matrix preserved from the previous step is dubbed “old” and hits from this matrix are assigned to be “true” for the current iteration. Respectively, the matrix employed at the current step plays the role of “new”. So, TP computes as the number of sites (hits) positively identified by the “new” matrix in the given functional window of length *L*; FP is the difference between the number of all sites detected by the new matrix in the functional window at all considered promoter sequences and TP. FN is the difference between the number of sites positively identified by the old matrix in the given functional window and TP. TN is the complement of sum of TP, FP and FN to the total expected number of all sites in the average given interval of the length *L* for the whole set of aligned promoter sequences (3).

The final result of this procedure is a PWM with optimized length and cut-off value *c_opt_*. The aforementioned refinement process is continued either until *Cor = 1* first time in the cycle or it reaches its maximum (usually 3 to 8 cycles). Each cycle brings a portion of new putative TFBSs overrepresented in this particular window and excludes some non-typical TFBSs. Each cycle consequently increases the influence of “similar” sites from the functional window. This influence is strongly supported by the requirement of keeping the magnitude of correlation coefficient *Cor* at a higher level. For functional windows with equally strong signals, all aforementioned steps are tested for each window and the window with the highest optimized *Cor* is selected (an example is shown in Figure 5).

In cases where the selection of a functional window is ambiguous, based on the observation of z-score spatial distribution on the promoters, we performed additional computations with variable window sizes around the area of potential signal and selected the window with maximum value of *Cor* for the initial *<* ***W^0^*** *,c_0_>* to prevent a high number of noisy sites at the initial step. This sort of heuristic was used to estimate the initial cut-off value as well. Changing the length of the motif is a typical part of the optimization procedure and its processing was performed independently from those of the functional window.

## Supporting Information

File S1 **Summary of predictions on synthetic tests.** Table S1, Summary from synthetic JASPAR tests with additions about performance of initial PWMs. Column descriptions correspond to Table 2.1 and Table 2.2 in text; L∶Np – length and number of promoter sequences in the test respectively. Table S2, Sequences with (T = C)GAGCG motif for TF Zeste.
(DOC)

File S2 **Sequence Logos for discovered putative TFBSs with JASPAR.** Includes four pages with complete sequence Logos for TF entries from TRANSFAC database, OPT mononucleotide PWMs, OPT dinucleotide PWMs and JASPAR which were not included in Figure 1 in the text.
(PDF)

File S3 **Sequence Logos for discovered putative TFBSs without JASPAR.** Contains the rest of optimized TF TRANSFAC entries which do not have JASPAR profiles to compare with, such as column D in Figure 1.
(PDF)

File S4 **The refined PWM in mono and dinucleotide formats.** Contains optimized PWMs.
(TXT)
