# Supplementary material for: Optimized Position Weight Matrices in Prediction of Novel Putative Binding Sites for Transcription Factors in the Drosophila melanogaster Genome
Source: PLoS One. 2013 Aug 6;8(8):e68712. doi: 10.1371/journal.pone.0068712 (PMC3735551; doi:10.1371/journal.pone.0068712)
Supplement: File S1 — Summary of predictions on synthetic tests. Table S1, Summary from synthetic JASPAR tests with additions about performance of initial PWMs. Column descriptions correspond to Table 2.1 and Table 2.2 in text; L∶Np – length and number of promoter sequences in the test respectively. Table S2, Sequences with (T = C)GAGCG motif for TF Zeste. (DOC) [file pone.0068712.s001.doc]

**Table S1. Summary from synthetic JASPAR tests with additions about performance of initial PWMs**. Column descriptions correspond to Table 2.1 and Table 2.2 in text; L:Np – length and number of promoter sequences in the test respectively.

| TF names | JASPAR test files | Initial PWM | | OPT mono PWM | | OPT di.  PWM | | Test size  L:Np |
| --- | --- | --- | --- | --- | --- | --- | --- | --- |
|  |  | TP | FP | TP | FP | TP | FP |  |
| Abd A  AP Q6  DEAF 01  E74A  En  Hb  KNI  KR Q6  PRD  Sn  SuH  Tll  Ubx  Z | MA0206.1.sites  MA0209.1.sites  MA0185.1.sites  MA0026.1.sites  MA0220.1.sites  MA0049.1.sites  MA0451.1.sites  MA0452.1.sites  MA0239.1.sites  MA0086.1.sites  MA0085.1.sites  MA0459.1.sites  MA0094.2.sites  MA0255.1.sites | 3  1  3  11  0  15  11  19  0  0  10  2  2  0 | 1  3  11  0  20  11  0  7  7  0  0  33  18  16 | 4  0  1  11  4  16  2  15  2  2  10  29  14  13 | 3  3  3  0  7  31  0  0  7  0  0  1  17  1 | 8  0  2  0  8  11  0  11  1  0  10  31  14  10 | 20  0  7  0  0  17  0  0  5  0  0  14  13  0 | 29:23  27:20  113:10  46:17  27:23  30:16  36:26  33:31  67:37  42:40  61:10  64:34  28:20  76:41 |
| TOTAL: | | 77 | 127 | 123 | 73 | 106 | 76 | 348 |

**Table S2. Sequences with (T=C)GAGCG motif for TF Zeste.**

| Found mono OPT | Found di OPT | TRANSFAC for Zeste (Z) |
| --- | --- | --- |
| AGTTGAGCG  ATTCGAGCG  CGTCGAGCG  CTCCGAGCG  GCTTGAGCG  GGTCGAGCG  GTTTGAGCG  TGCCGAGCG  TTTGGAGCG | AGTTTGAGCGC  CAGTTGAGCGC  CGGTCGAGCGG  CTTTGGAGCGA  TAGCTGAGCGG  TTTGCGAGCGG  TCGTCGAGCGG | TCACTGAGCGA  TTATTGAGCGG  TTTTTGAGCGC  GTTTTGAGCGT |
