# Supplementary figures and images for: Optimized Position Weight Matrices in Prediction of Novel Putative Binding Sites for Transcription Factors in the Drosophila melanogaster Genome
Source: PLoS One. 2013 Aug 6;8(8):e68712. doi: 10.1371/journal.pone.0068712 (PMC3735551; doi:10.1371/journal.pone.0068712)

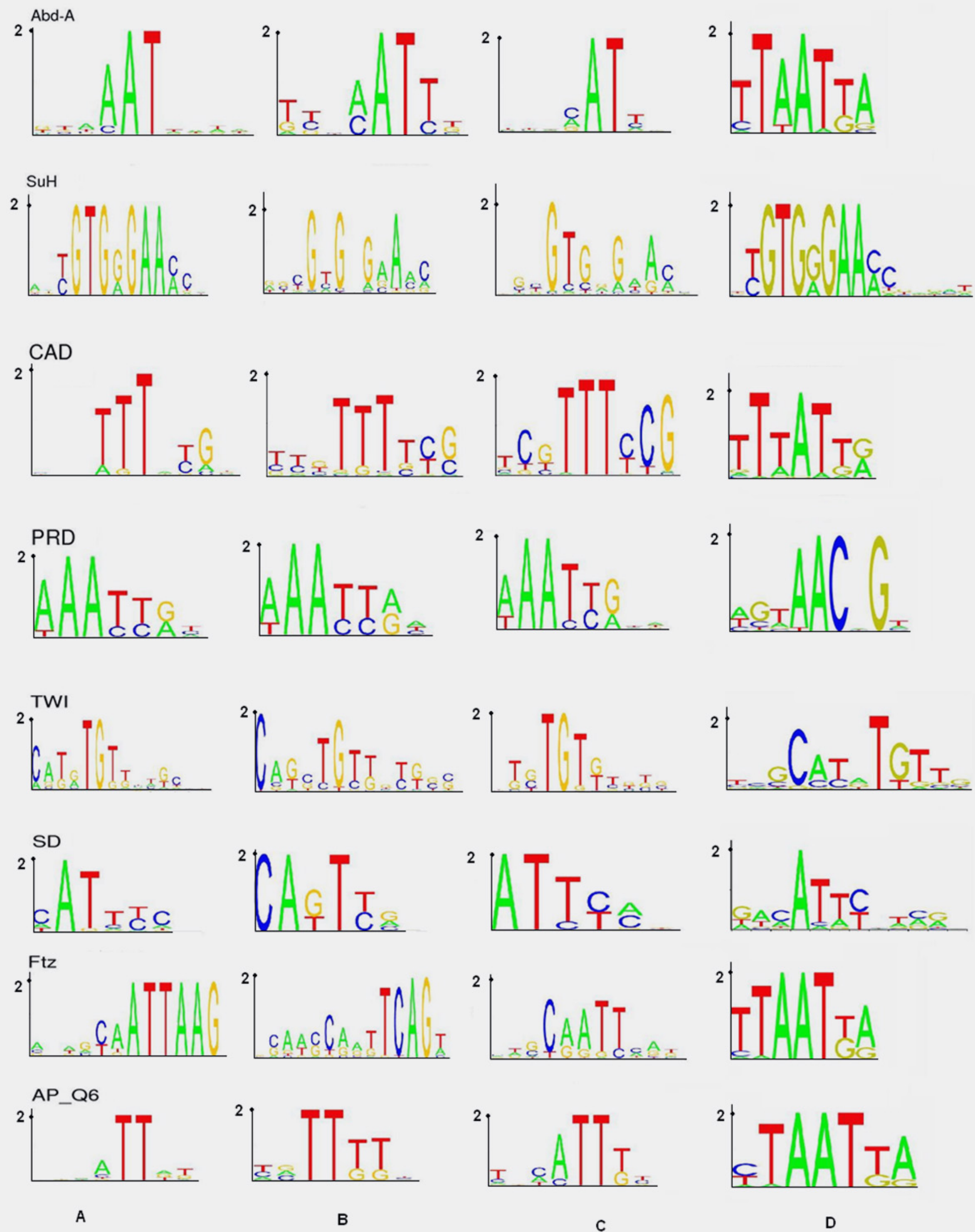

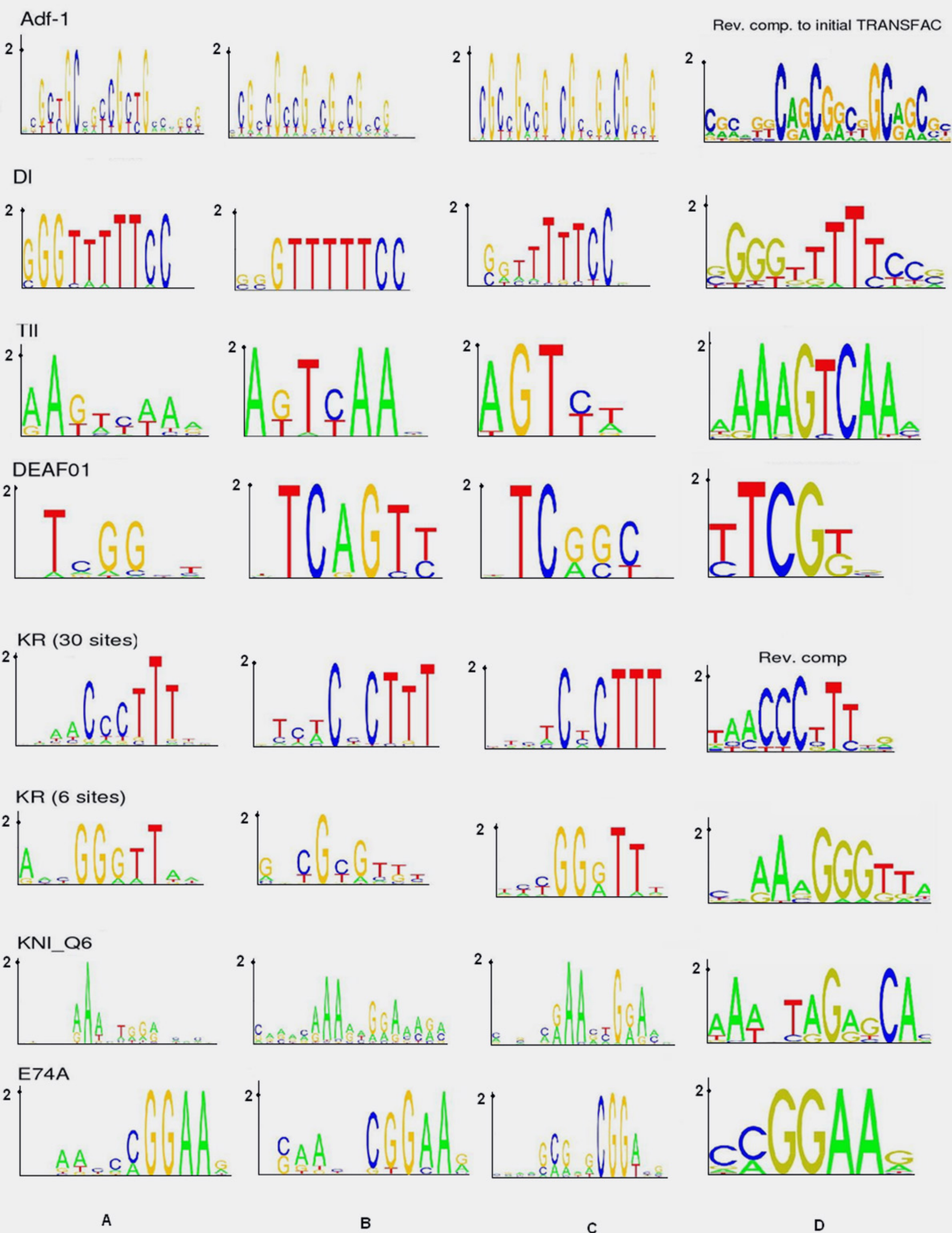

Sn 22 sites

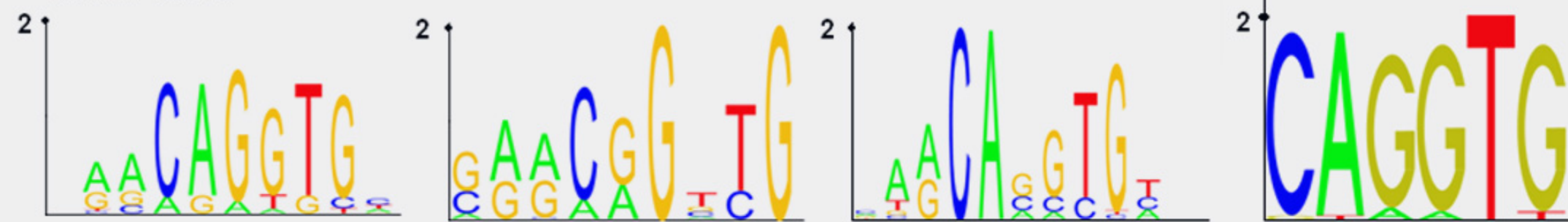

Sn 12 sites

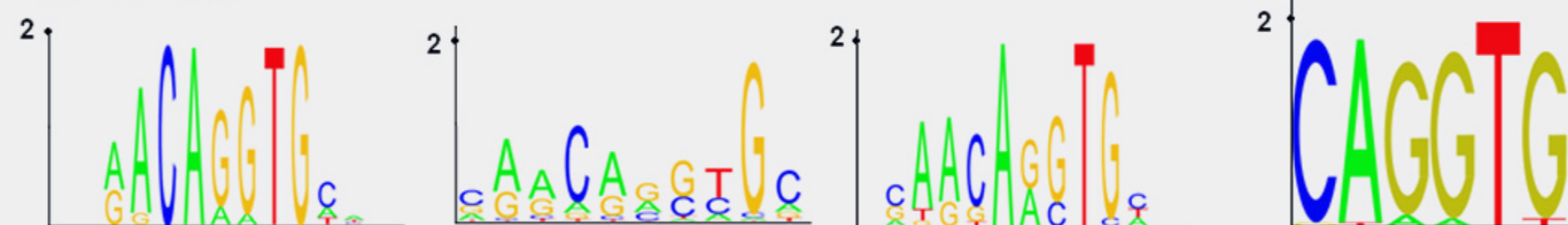

Ovo 9 sites

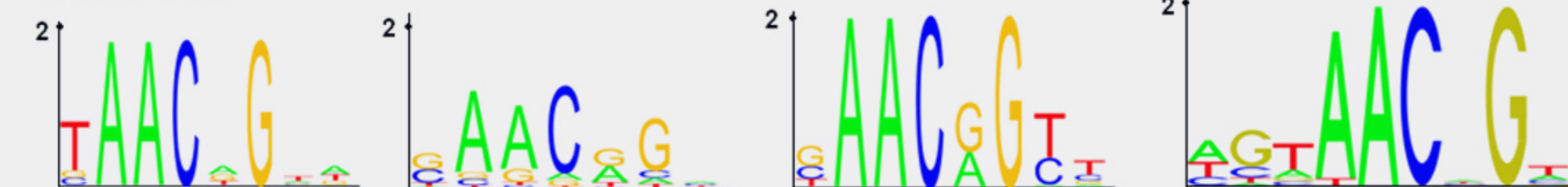

Ovo 21 sites

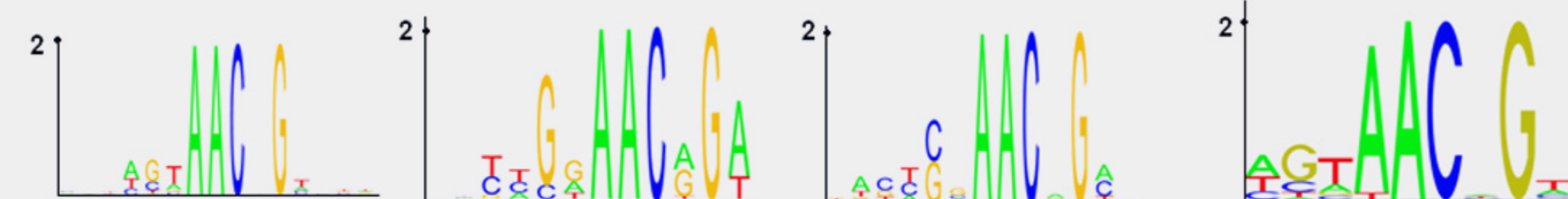

Hb

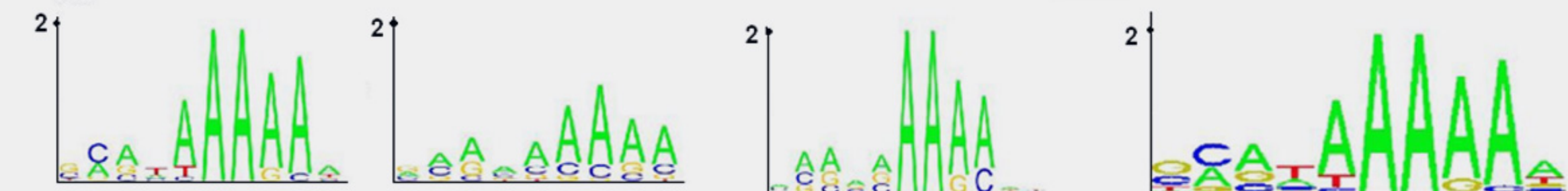

A

B

C

D

Supplement: File S2 — Sequence Logos for discovered putative TFBSs with JASPAR. Includes four pages with complete sequence Logos for TF entries from TRANSFAC database, OPT mononucleotide PWMs, OPT dinucleotide PWMs and JASPAR which were not included in Figure 1 in the text. (PDF) [file pone.0068712.s002.pdf]

Sn (9 sites)

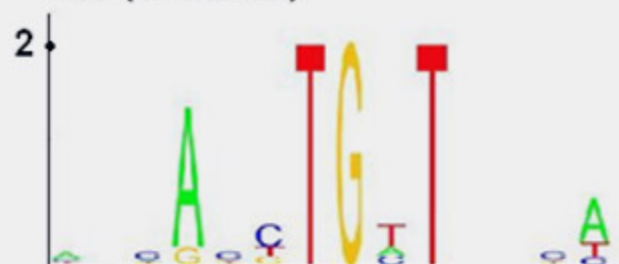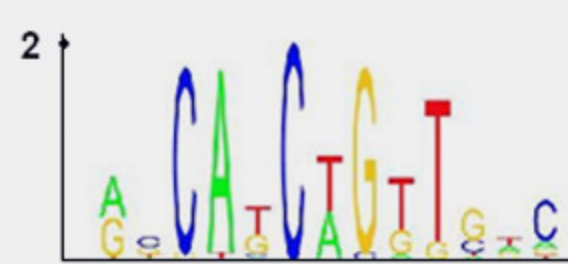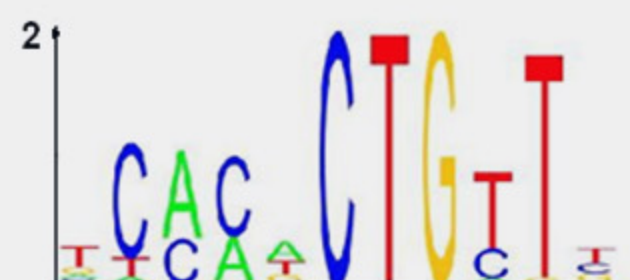

CF1A

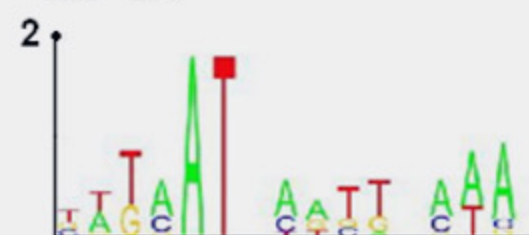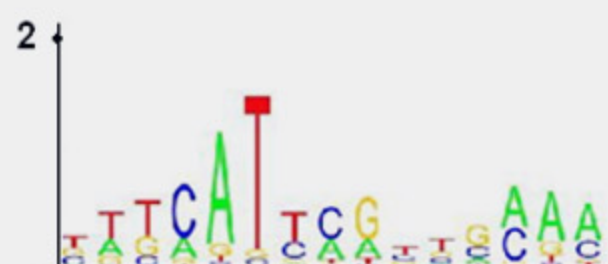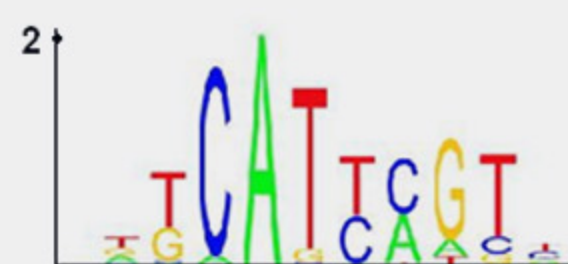

Sry beta

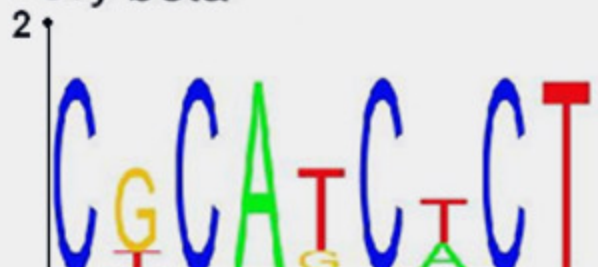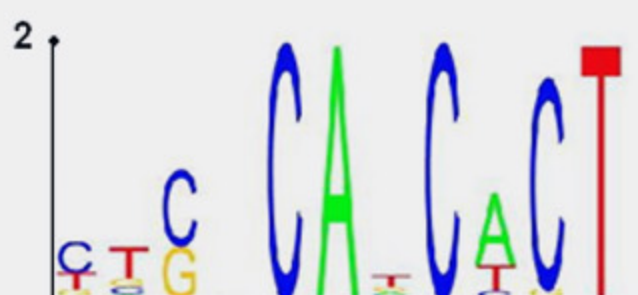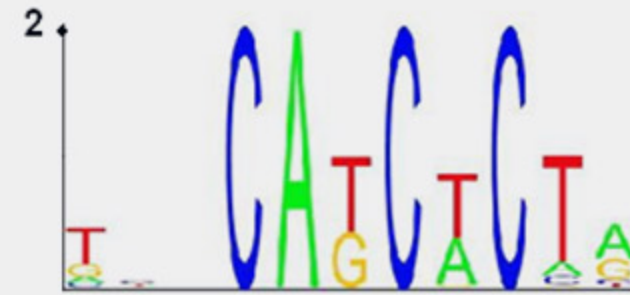

TCF

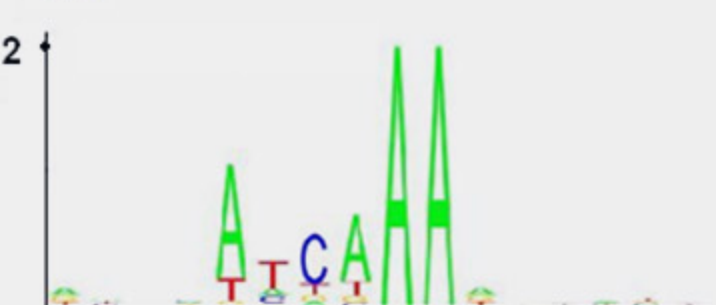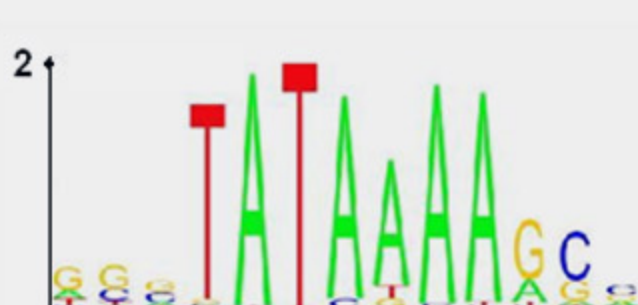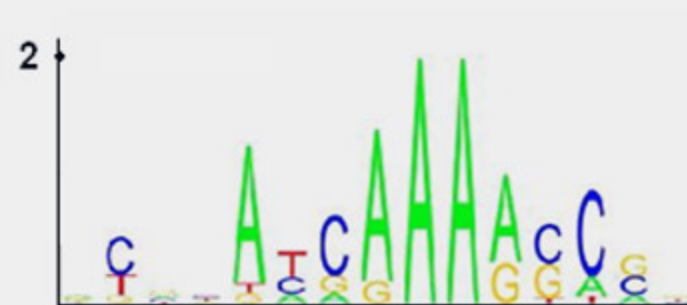

C/EBP

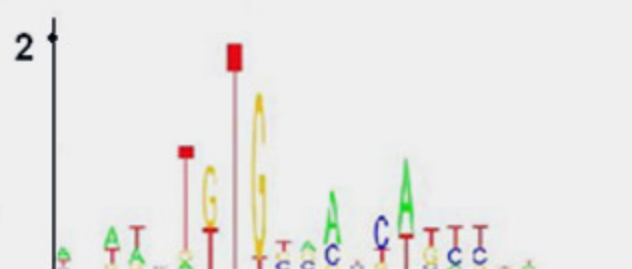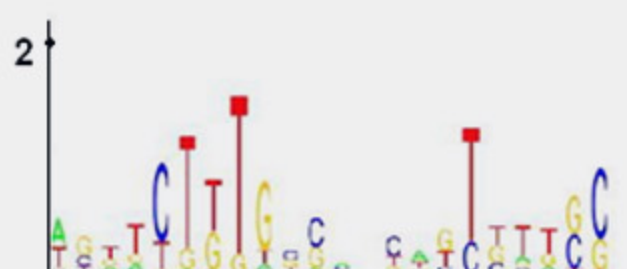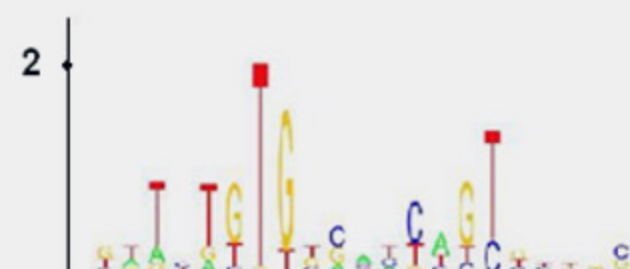

CF1\_02

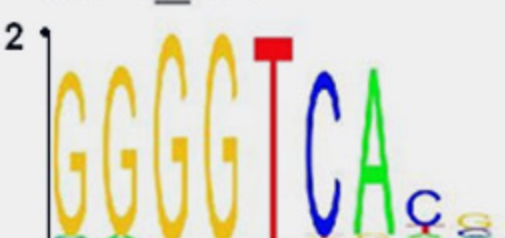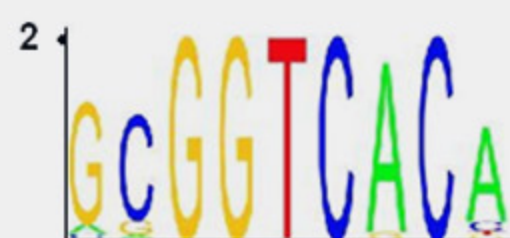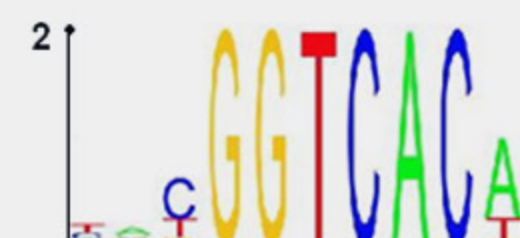

DREF\_Q3

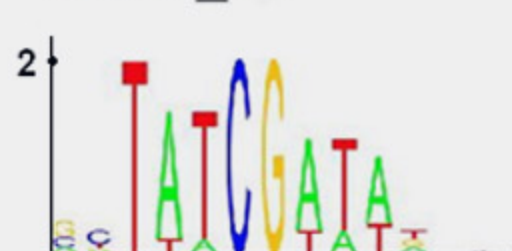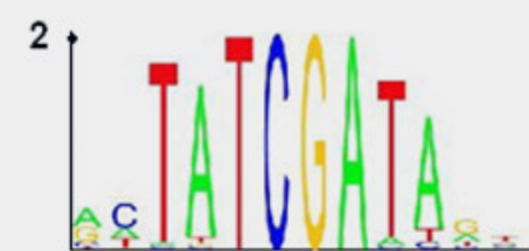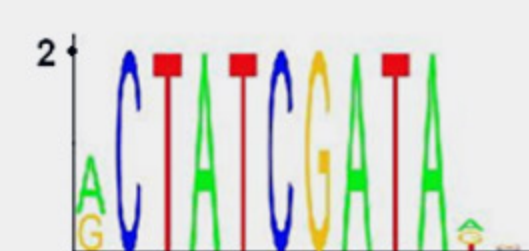

A

B

C

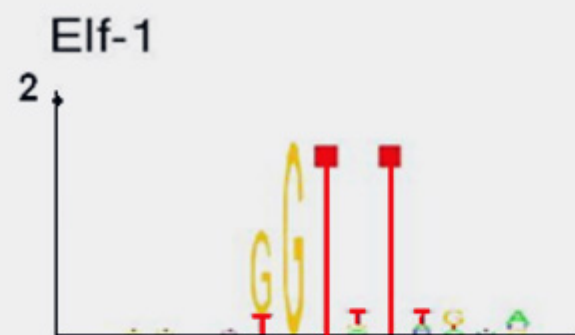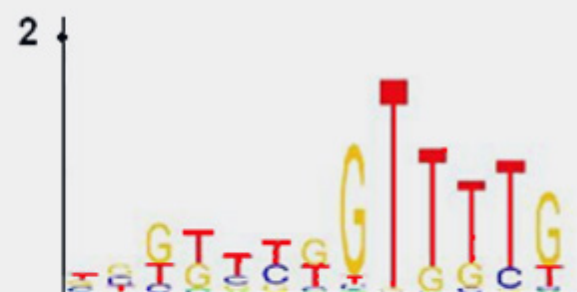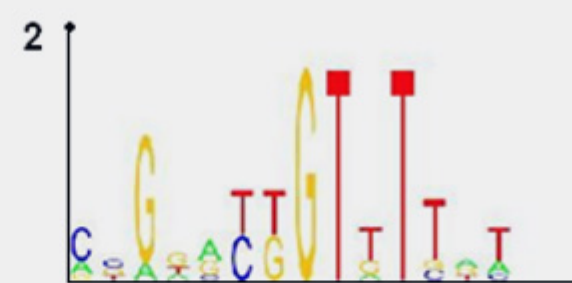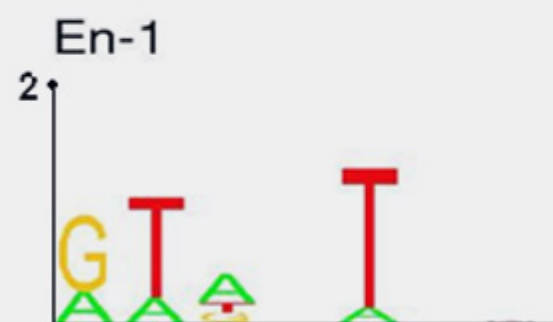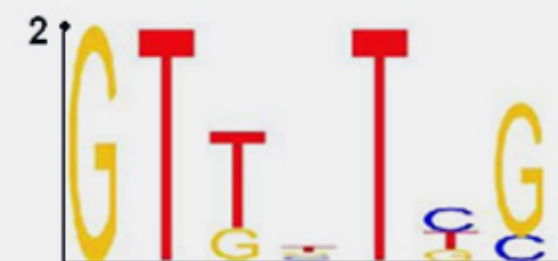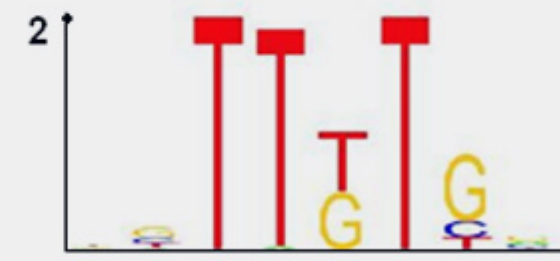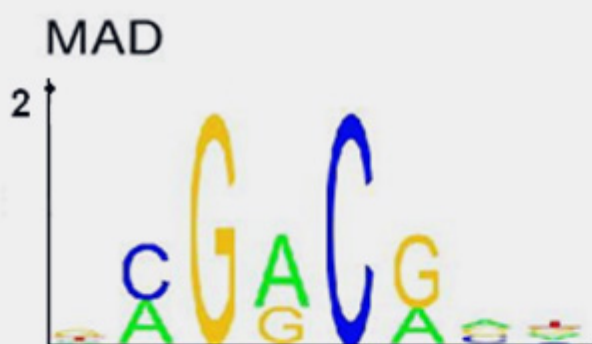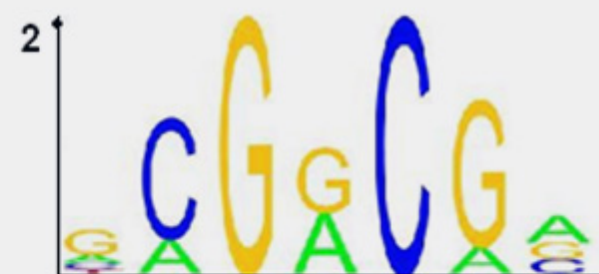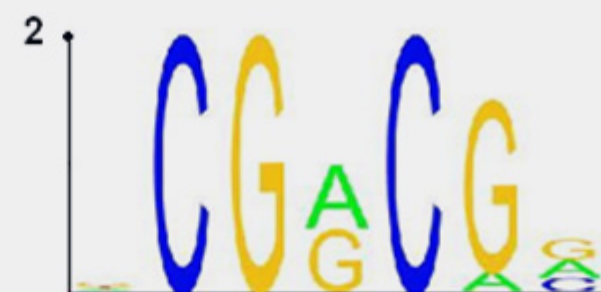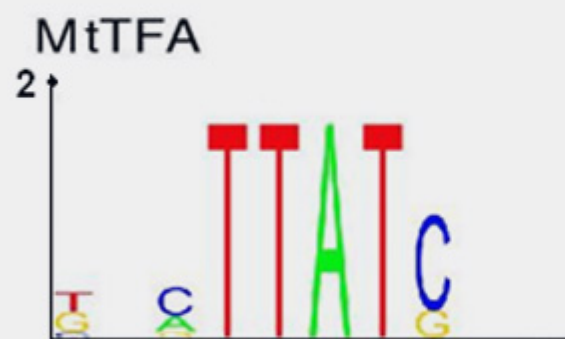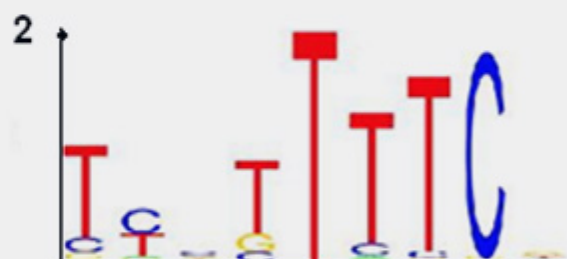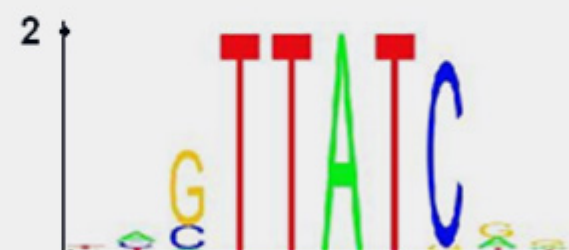

A

B

C

Supplement: File S3 — Sequence Logos for discovered putative TFBSs without JASPAR. Contains the rest of optimized TF TRANSFAC entries which do not have JASPAR profiles to compare with, such as column D in Figure 1. (PDF) [file pone.0068712.s003.pdf]
